# Comparative Proteomic Analysis of Differential Responses of *Pinus massoniana* and *Taxus wallichiana* var*. mairei* to Simulated Acid Rain

**DOI:** 10.3390/ijms15034333

**Published:** 2014-03-12

**Authors:** Wen-Jun Hu, Juan Chen, Ting-Wu Liu, Martin Simon, Wen-Hua Wang, Juan Chen, Fei-Hua Wu, Xiang Liu, Zhi-Jun Shen, Hai-Lei Zheng

**Affiliations:** 1 Key Laboratory for Subtropical Wetland Ecosystem Research of MOE, College of the Environment and Ecology, Xiamen University, Xiamen 361005, Fujian, China; E-Mails: huwenjun@stu.xmu.edu.cn (W.-J.H.); chenjuanmn@xmu.edu.cn (J.C.); liutw@xmu.edu.cn (T.-W.L.); martinsimon23@gmail.com (M.S.); wangwenhua107@xmu.edu.cn (W.-H.W.); chenjuan2008@xmu.edu.cn (J.C.); wufeihua@xmu.edu.cn (F.-H.W.); lx19890818@stu.xmu.edu.cn (X.L.); shenzj456@sina.com (Z.-J.S.); 2 Department of Biology, Huaiyin Normal University, Huaian 223300, Jiangsu, China; 3 College of Life and Environmental Sciences, Hangzhou Normal University, Hangzhou 310036, Zhejiang, China

**Keywords:** acid rain tolerance, proteomic, *Pinus massoniana*, stress response, *Taxus wallichiana* var*. mairei*, woody plant

## Abstract

Acid rain (AR), a serious environmental issue, severely affects plant growth and development. As the gymnosperms of conifer woody plants, *Pinus massoniana* (AR-sensitive) and *Taxus wallichiana* var*. mairei* (AR-resistant) are widely distributed in southern China. Under AR stress, significant necrosis and collapsed lesions were found in *P. massoniana* needles with remarkable yellowing and wilting tips, whereas *T. wallichiana* var*. mairei* did not exhibit chlorosis and visible damage. Due to the activation of a large number of stress-related genes and the synthesis of various functional proteins to counteract AR stress, it is important to study the differences in AR-tolerance mechanisms by comparative proteomic analysis of tolerant and sensitive species. This study revealed a total of 65 and 26 differentially expressed proteins that were identified in *P. massoniana* and *T. wallichiana* var*. mairei*, respectively. Among them, proteins involved in metabolism, photosynthesis, signal transduction and transcription were drastically down-regulated in *P. massoniana*, whereas most of the proteins participating in metabolism, cell structure, photosynthesis and transcription were increased in *T. wallichiana* var*. mairei*. These results suggest the distinct patterns of protein expression in the two woody species in response to AR, allowing a deeper understanding of diversity on AR tolerance in forest tree species.

## 1. Introduction

Constant increase of human activities in recent decades results in some regions suffering from environmental pollution, such as acid rain (AR), especially in the fast developing regions of southern China [1,2]. To date, AR continues to threaten many sensitive ecosystems and cause detrimental impacts on local vegetation [3,4]. For instance, AR can induce necrosis and chlorosis in leaves of AR-sensitive plants and even structural abnormalities through damaging the cuticles of the epidermis and leaf mesophyll cells [5,6]. In addition, AR can also disturb the normal metabolism of plants and cause a decrease in photosynthesis, depression in plant growth and development, and even the death of plants in some extreme cases [7].

*Pinus massoniana*, a widely distributed coniferous species in southern China, has suffered seriously from AR in recent decades [8]. Severe defoliation of *P. massoniana* in several AR-affected regions of southern China was revealed in previous studies and the growth was significantly reduced under AR treatment [9,10]. *Taxus wallichiana* var*. mairei*, another conifer tree species, is distributed in adjacent regions that are also suffering from serious AR [11]. Physiological and biochemical changes and growth responses to AR have been reported in *T. wallichiana* var. *mairei*. The results suggest that *T. wallichiana* var. *mairei* is a resistant species to AR stress [12,13]; however the biochemical and physiological features and molecular mechanisms of such a distinction remains poorly understood. Analyzing protein expression changes under AR stress is a powerful way to reveal the molecular mechanism through a comparison between AR-sensitive and AR-resistant species.

Recently, by using a 2-DE-based proteomic approach, a set of proteins associated with: primary metabolism, secondary metabolism, protein stability and defense, photosynthesis and energy metabolism, *etc*., have been successfully identified and characterized, and may play important roles in mediating plant responses to AR for *P. massoniana* and *Arabidopsis* [14–16]. In the present study, a 2-DE and MALDI-TOF MS study was performed to identify the global changes in protein expression under AR treatment in *P. massoniana* and *T. wallichiana* var*. mairei*, which respectively represent AR-sensitive and AR-tolerant species. Furthermore, we also characterized the AR-responsive proteins, analyzed the functions of these differentially expressed proteins, and established the different responsive networks of metabolism in *P. massoniana* and *T. wallichiana* var*. mairei* under AR stress.

The results of this study, providing different evidence from that evaluating the effects of AR stress on single tree species, could further enhance the current understanding of the protein changes underlying AR stress-related cellular and physiological responses between AR-sensitive and AR-resistant woody plants, and further dissect the different tolerance mechanisms of forest trees to AR stress.

## 2. Results and Discussion

### 2.1. Results

#### 2.1.1. Phenotype and Physiological Changes in *P. massoniana* and *T. wallichiana* var*. mairei* under AR

**Figure 1.**
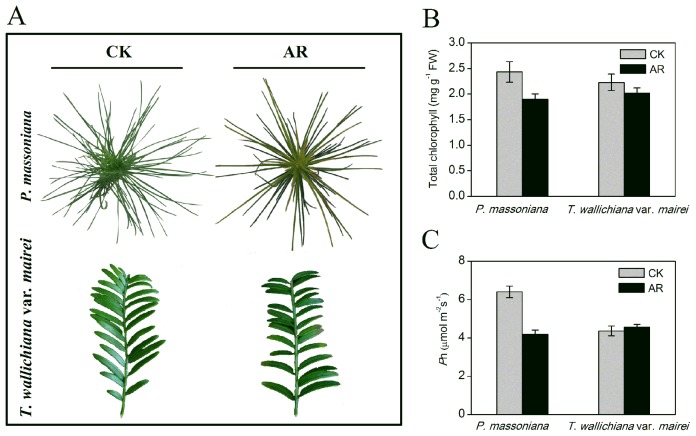
Morphological and physiological changes of *P. massoniana* and *T. wallichinana* var. *mairei* under distilled water as control (CK) and simulated acid rain (AR) stress. (**A**) Injury phenotype; (**B**) total chlorophyll content; (**C**) photosynthesis (*P*n). Data are means ± SE from measurements of five replicate experiments.

In order to study the responses of woody plant to AR stress, *P. massoniana* and *T. wallichiana* var. *mairei*, an AR-sensitive and AR-tolerant tree species respectively, were treated with simulated AR (pH 3.0) for two months. First we investigated the detrimental phenotypical and physiological changes of these two species under AR stress. Morphological changes of both species are shown in Figure 1A, the chlorophyll content and net photosynthetic rate (*P*n) of the saplings were also measured (Figure 1B,C). After two months of AR treatment, the significant necrosis and collapsed lesions were found in needles with remarkable yellowing and wilting tips in *P. massoniana*, whereas *T. wallichiana* var*. mairei* did not exhibit chlorosis and visible damage (Figure 1A). As shown in Figure 1B,C, the chlorophyll content and *P*n were significantly decreased in *P. massoniana* under simulated AR treatment. Similar results were observed in AR-treated *Arabidopsis* and woody plants [10,14,17]. On the contrary, no significant changes in the chlorophyll content and *P*n were found in *T. wallichiana* var*. mairei* (Figure 1B,C) under simulated AR, indicating that *T. wallichiana* var*. mairei* is more resistant than *P. massoniana* in response to AR stress.

#### 2.1.2. Identification and Functional Classification of AR-Responsive Proteins in *P. massoniana* and *T. wallichiana* var. *mairei*

**Figure 2.**
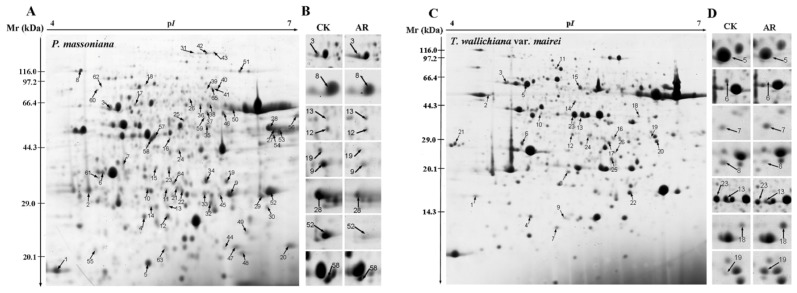
2-DE image analysis of proteins extracted from *P. massoniana* and *T. wallichinana* var. *mairei* leaves. The numbers assigned to the proteins spots correspond to those listed in Tables 1 and 2. (**A**) A representative Coomassie Brilliant Blue (CBB) R250-stained 2D gel of total soluble proteins from *P. massoniana*. Arrows indicate 65 spots showing at least 2-fold changes (*p* < 0.05) were analyzed by Matrix-assisted laser desorption/ionization-time-of-flight mass spectrometry (MALDI-TOF MS); (**B**) Close-up view of some differentially expressed protein spots in *P. massoniana*; (**C**) A representative CBB R250-stained 2D gel of total soluble proteins from *T. wallichinana* var. *mairei*. Arrows indicate 26 spots showing at least 2-fold changes (*p* < 0.05) were analyzed by MALDI-TOF MS; (**D**) Close-up view of some differentially expressed protein spots in *T. wallichinana* var. *mairei*.

To further explore the proteome changes in *P. massoniana* and *T. wallichiana* var*. mairei* leaves under AR treatment, 2-DE was performed in this study. A total of 73 protein spots from *P. massoniana* gels and 31 protein spots from *T. wallichiana* var*. maire* gels showed significant changes. Tables S1 and S2 showed the details of identified proteins. Out of these spots, a total of 65 and 26 proteins were identified by mass spectrometry in *P. massoniana* and *T. wallichiana* var. *mairei*, respectively. Those with expression abundance changed more than 2-fold among three different repetitions, were identified as the differentially expressed proteins after AR treatment (Figure 2). Close-up views of several protein spots are shown in Figure 2B,D.

**Figure 3.**
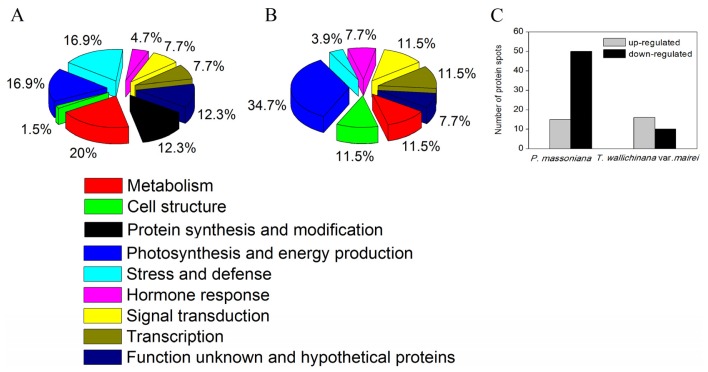
Functional category distribution of the identified proteins in *P. massoniana* and *T. wallichinana* var. *mairei* under the AR treatment. Each identified protein listed in Tables 1 and 2 was functionally classified based on their known and putative functions. The pie chart indicates the percentage of the AR-responsive proteins identified in each functional category. (**A**) AR-responsive proteins in *P. massoniana*; (**B**) AR-responsive proteins in *T. wallichinana* var. *mairei*; (**C**) Number of protein spots significantly up-regulated and down-regulated in *P. massoniana* and *T. wallichinana* var. *mairei* under AR stress.

The identified proteins were divided into seven groups based on their biological functions in both *P. massoniana* and *T. wallichiana* var*. mairei* (Figure 3A,B). In *P. massoniana*, the largest group was metabolism (20%), the following groups were photosynthesis and energy production (16.9%), stress and defense (16.9%), protein synthesis and modification (12.3%), signal transduction (7.7%), transcription (7.7%), hormone response (4.7%), cell structure (1.5%), and function unknown and hypothetical proteins (12.3%) (Figure 3A). In *T. wallichiana* var*. mairei*, the identified proteins were involved in photosynthesis and energy production (34.7%), metabolism (11.5%), cell structure (11.5%), signal transduction (11.5%), transcription (11.5%), stress and defense (3.9%), hormone response (7.7%), and function unknown and hypothetical proteins (7.7%) (Figure 3B). This result suggests that proteins involved in metabolism, cell structure, protein synthesis-related proteins, stress response, signal transduction and transcription play an important role in AR tolerance.

**Figure 4.**
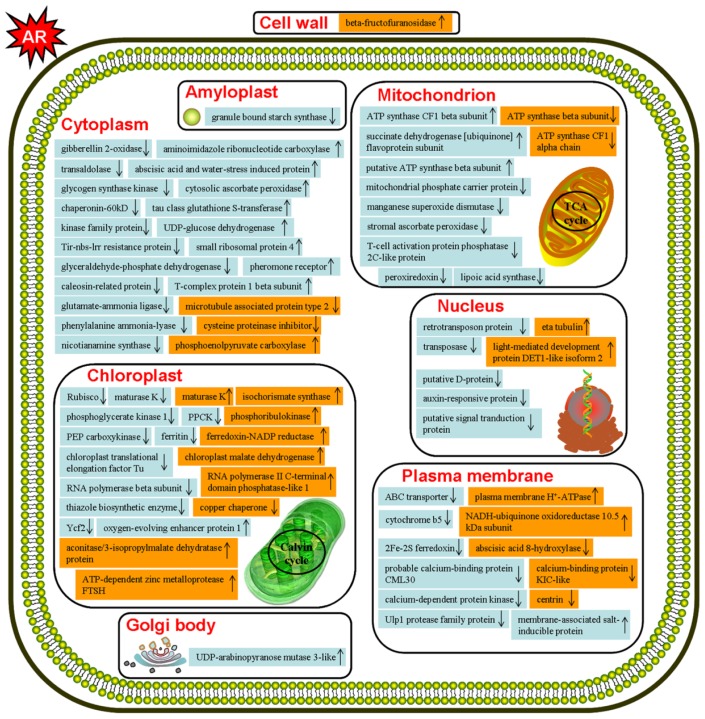
Putative subcellular location model of AR stress response in *P. massoniana* and *T. wallichinana* var. *mairei*. The proteins marked in blue or orange represents they were found in *P. massoniana* or *T. wallichinana* var. *mairei*, respectively. Some of the AR-responsive proteins are indicated with those up-regulated marked by ↑ and those down-regulated marked by ↓. The abbreviations used in the Figure were explained in Tables 1 and 2.

As shown in Figure 3C, the number of differentially expressed protein spots was more than double in *P. massoniana* compared to those in *T. wallichiana* var*. mairei* under AR stress. In AR-treated *P. massoniana*, 15 proteins were increased and 50 proteins were decreased (Figure 3C). Interestingly, 16 proteins were increased and only 10 proteins were decreased in AR-treated *T. wallichiana* var*. mairei* (Figure 3C). Most members of protein families were expressed in cytoplasm, chloroplasts, mitochondria and plasma membrane (Figure 4). After AR treatment, some proteins in *T. wallichiana* var*. mairei* were found to exhibit different expression patterns compared to *P. massoniana*, suggesting that tolerant plants can equip themselves better to respond to AR stress by provoking related proteins expression.

### 2.2. Discussion

#### 2.2.1. Metabolism Related Proteins

Environmental stresses severely affect the metabolism of plants [15]. Thirteen metabolism-related proteins were exclusively affected by AR stress, with eleven down-regulated and two up-regulated in *P. massoniana* (Table 1) in our study. As observed in *T. wallichiana* var*. mairei*, only four up-regulated and two down-regulated proteins involved in metabolism were identified with altered abundance at least two-fold in response to AR treatment (Table 2). Nitrogen is an essential building block of nucleic acids and proteins, and nitrate assimilation greatly contributes to plant primary productivity [18]. Previous studies showed that salinity and water deficit strongly affected nitrogen metabolism and uptake of nitrate in wheat and rice [19,20]. In our study, glutamate-ammonia ligase, a key enzyme for nitrogen metabolism, belonging to the glutamine synthetase family was identified [18]. Kong *et al*. reported that low pH could potentially decrease the efficiency of nitrogen availability in *P. massoniana* [8]. Consistent with previous results, the abundance of glutamate-ammonia ligase (spot 28, Table 1) was decreased in *P. massoniana* under AR stress indicating that AR affected the primary metabolism of nitrogen in *P. massoniana*, whereas no nitrogen metabolism-related proteins have been identified in *T. wallichiana* var*. mairei*.

Donation of electrons by ferredoxin (Fd) has been demonstrated in many other plastid enzymes, which is essential for multiple cellular processes, including nitrogen and sulfur assimilation, amino acid and fatty acid synthesis [21,22]. In this study, the abundance of 2Fe-2S ferredoxin (spot 48, Table 1) was decreased in AR-treated *P. massoniana*, indicating that nitrogen metabolism again, as well as sulfur metabolism, may be affected by AR treatment. Ferredoxin (flavodoxin)-NADP(H) reductases (FNRs) are ubiquitous flavoenzymes that deliver NADPH or low potential one-electron donors (ferredoxin) to redox-based metabolisms in plastids and mitochondria [23]. It is noteworthy that the overexpression of FNR can increase tolerance to oxidative stress in transgenic tobacco plants [24]. The up-regulation of FNR (spot 9, Table 2) in response to AR stress in *T. wallichiana* var*. mairei* reflects the role of this protein in basal metabolism under stress conditions. ABC transporters constitute one of the largest protein families with diverse functions in membrane transport [25]. Compared with control treatment, the abundance of ABC transporter (spot 39, Table 1) was decreased in *P. massoniana* under AR treatment. Earlier studies have detected a decrease in ABC transporter substrate binding protein in response to copper stress in *Cannabis sativa* roots, suggesting that the ABC transporter may play a very important role in the tolerance response of plant to environmental stresses [26]. Furthermore, the importance of ABC transporters has been reported for the integration of mitochondria in plant cellular iron homeostasis [25]. In plants, ferritin is an essential regulator of iron homeostasis, and the gene expression of ferritin is modulated by many environmental factors including drought and cold [27]. In our study, the abundance of ferritin (spot 57, Table 1) was decreased in *P. massoniana* under AR stress. The down-regulation of the protein related to transmembrane transport of molecules (e.g., the ABC transporter) and down-regulation of metal ion related proteins, is propitious for re-establishing cellular homeostasis in AR-treated *P. massoniana*. Based on the above results, we speculate that AR stress might affect more metabolic processes in *P. massoniana* than those in *T. wallichiana* var*. mairei*.

#### 2.2.2. Cell Structure Related Proteins

The cytoskeleton is rapidly remodeled by various endogenous and external stimuli. We found that microtubule associated protein type 2 (spot 23, Table 2) decreased in abundance following AR treatment, while eta tubulin (spot 25, Table 2) was increased in *T. wallichiana* var*. mairei* under AR stress. Previous studies reported that the transverse orientation of cortical microtubule arrays in tobacco BY-2 cells was remodelled to a more random arrangement and the tubulin a-6 chain was induced in *Arabidopsis* roots after NaCl treatment [28]. Our results support that the accumulation of microtubule associated protein, rather than eta tubulin, could play a crucial role in the resistance to AR stress in *T. wallichiana* var. *mairei*. These universal cytoskeletal proteins may call into question their stronger tolerance in *T. wallichiana* var. *mairei* in response to AR than that in *P. massoniana*. Cell wall proteins are essential constituents of the plant cell wall, which are involved in modifications of cell wall components and structure, and signaling and interactions with plasma membrane proteins. Beta-fructofuranosidase is an enzyme involved in cell wall biosynthesis. In this study, the abundance of beta-fructofuranosidase (spot 14, Table 2) was increased in *T. wallichiana* var*. mairei* under AR treatment, indicating the stronger capacity of *T. wallichiana* var*. mairei* to recover from AR stress. In addition, UDP-glucose dehydrogenase, which is involved in cell wall pectin metabolic process, greatly contributes to AR-induced cell wall rigidification and physical barrier formation. The abundance of UDP-glucose dehydrogenase (spot 62, Table 1) was down-regulated in *P. massoniana* under AR treatment, which may result in perturbation of cell wall structure and more seriously damaged phenotypes in AR-treated *P. massoniana*.

#### 2.2.3. Protein Synthesis and Modification Related Proteins

It is not surprising that AR stress also damaged the homeostasis of protein metabolism between biosynthesis and degradation. Singh *et al.* found that chloroplast translation elongation factor (EF-Tu) could play an important role in plant adaptation to environmental stresses in addition to its role in peptide elongation [29]. Indeed, our results provided additional evidence that a chloroplast translational elongation factor Tu (spot 31, Table 1) was down-regulated in *P. massoniana* after AR treatment, indicating that protein synthesizing machinery plays an important role in AR adaptation in this plant. Glycogen synthase kinase 3 (GSK-3) was originally identified as a regulator of glycogen synthesis in mammals. In plants, GSKs are reported to be involved in diverse important processes including hormone signaling, development, pathogenic stimuli and stress responses [30]. In this study, a glycogen synthase kinase (spot 45, Table 1) was down-regulated in *P. massoniana* under AR treatment. Indeed, our result provides extra evidence that GSKs play an important regulatory function under AR stress. Moreover, it is known that chaperonin is down-regulated under oxidative stress in rice [31]. Consistent with previous results, the decreased abundance of chaperonin-60kD (spot 49, Table 1) was observed in AR-treated *P. massoniana*. This finding suggests that chaperonin may be a general AR stress response element in *P. massoniana*. As a whole, these results suggest that AR treatment affected the biosynthesis and refolding of proteins and led to protein degradation, which is more pronounced in *P. massoniana* than in *T. wallichiana* var*. mairei*.

#### 2.2.4. Photosynthesis and Energy Production Related Proteins

Photosynthesis is a key plant process affected by many environmental stresses. Physiological analysis showed the attenuation of photosynthesis in AR-stressed *P. massoniana* leaves, and similar patterns were also observed for most photosynthesis-related proteins (ribulose-1,5-bisphosphate carboxylase/oxygenase large subunit (Rubisco) (spots 44, 50, 55 and 60, Table 1), phosphoglycerate kinase (spot 53, Table 1)), whose abundances were deceased in AR-treated *P. massoniana*. Ohta *et al*. found that Rubisco was decreased in *Synechocystis* sp. PCC 6803 under acid condition [32]. Consistent with our previous results, AR could lead to a remarkable decrease in the efficiency of photosynthesis in plants, and proteomic studies demonstrated that Rubisco was decreased under AR stress in *Arabidopsis* [10,15]. On the other hand, it has been reported that the transcription of Calvin cycle enzymes were decreased in drought- and salt-stressed barley [33]. In *P. massoniana*, phosphoglycerate kinase (spot 53, Table 1) was found to be down-regulated under AR treatment. These results suggest that the photosynthesis apparatus is susceptible to AR stress, which may be one of the major reasons for decreased chlorophyll content and photosynthesis under AR stress in *P. massoniana* (Figure 1B,C). On the contrary, no significant change in AR-stressed *T. wallichiana* var. *mairei* leaves (Figure 1B,C), photosynthesis-related proteins including NADH-ubiquinone oxidoreductase 10.5 kDa subunit (spot 1, Table 2), phosphoribulokinase (spot 10, Table 2) and ATP-dependent zinc metalloprotease FTSH (spot 11, Table 2) were increased in *T. wallichiana* var. *mairei* under AR treatment. For example, phosphoribulokinase that catalyzes the final step in the regeneration of ribulose-1,5-bisphosphate in the Calvin cycle, was up-regulated by AR in *T. wallichiana* var. *mairei* in this study, which was different from what was found in *P. massoniana*. On the other hand, the expression of ATP-dependent zinc metalloprotease FTSH (spot 11, Table 2), a membrane bound protein located in thylakoids involved in the removal of a damaged D1 protein from PSII in plants, was also up-regulated in AR-treated *T. wallichiana* var. *mairei*. These findings indicated that the mechanisms of photosynthetic metabolism under AR stress are different between *P. massoniana* and *T. wallichiana* var. *mairei*. Photosynthesis apparatus is susceptible to AR stress, which may be one of the major reasons for decreased chlorophyll content and photosynthesis under AR stress in *P. massoniana*. On the contrary, photosynthesis-related proteins were increased in *T. wallichiana* var. *mairei* under AR treatment, which may compensate the impaired photosynthesis apparatus by AR. Thus, no significant changes in the chlorophyll content and *P*n were found in *T. wallichiana* var*. mairei* under simulated AR treatment, indicating that *T. wallichiana* var*. mairei* is more resistant than *P. massoniana* in response to AR stress. We presumed that *T. wallichiana* var*. mairei* is an AR-tolerant species with a high capacity for regulating related proteins to enhance photosynthetic metabolism, and inhibit the impaired effect on photosynthesis by AR stress.

Different from photosynthesis, the accumulation of some energy production and conversion related proteins were increased in *P. massoniana* under AR treatment. Gao *et al.* reported that ATPase beta subunit and ATPase CF1 beta chain were up-regulated in leaves of wheat subjected to salt treatment [34]. Other evidence also indicates that sufficient ATP is necessary for plant growth, development and response to stress [28]. In *P. massoniana*, ATP synthase CF1 beta subunit (spot 3, Table 1), putative ATP synthase beta subunit (spot 17, Table 1) and succinate dehydrogenase (ubiquinone) flavoprotein subunit (spot 65, Table 1) were found to be up-regulated by AR stress in our study. These results indicated that more energy is required for reinforcing *P. massoniana* resistance to AR stress. On the contrary, energy conversion-related proteins including ATP synthase beta subunit (spots 3 and 5, Table 2) and ATP synthase CF1 alpha chain (spot 15, Table 2) showed lower expression in response to AR stress in *T. wallichiana* var*. mairei*, which suggests different mechanisms of energy production in *T. wallichiana* var*. mairei* exposed to AR treatment.

#### 2.2.5. Stress and Hormone Response Related Proteins

Reactive oxygen species (ROS), which play a critical role in plant cellular signaling and stress responses, are readily produced by abiotic stresses [35]. Plants can regulate the ROS level through complex mechanisms such as scavenging ROS with ascorbate peroxidase (APX), and glutathione S-transferase (GST). In this study, both APX and GST (spots 10, 13 and 14, Table 1) were increased in abundance in AR-treated *P. massoniana*. In addition, we found that several other enzymes associated with stress and defense were increased in abundance in *P. massoniana*, including membrane-associated salt-inducible protein (spot 51, Table 1). Thus, the up-regulated expression of these proteins implies that the antioxidative defense system was provoked in AR-treated *P. massoniana* seedlings, and such a consistent induction is likely a consequence of antioxidative reactions in plants under AR stress. We speculate that *P. massoniana* needs to provoke more defense and stress related proteins against AR stress, and does not have similar protein accumulation such as that found in *T. wallichiana* var*. mairei* (AR-resistant). These results, together with the complex expression patterns of stress related proteins in both woody plants provide new insights into the relationships between the impacts of AR stress and defense and stress responses.

Plant hormones are not only involved in plant growth and development, but also important in response to abiotic and biotic stresses. Recent studies suggest that environmental stimuli can regulate endogenous gibberellin (GA) level through the changes of enzymes involved in GA biosynthesis and inactivation [36]. In this study, the abundance of gibberellin 2-oxidase (spot 40, Table 1) decreased in *P. massoniana* under AR stress, which is consistent with our previous results that AR stress led to a decrease in the abundance of gibberellin-responsive protein in *Arabidopsis* [15], suggesting the important roles of gibberellin 2-oxidase and GA in AR tolerance in *P. massoniana*. Moreover, the abundance of auxin-responsive protein (spot 47 Table 1) decreased in AR-treated *P. massoniana*, suggesting that the auxin pathway may play a role in mediating AR-sensitive woody plants responses to AR stress. Isochorismate synthase (ICS) is required to synthesize salicylic acid for plant defense [37]. Furthermore, it has been reported that the salicylic acid signaling pathway is implicated in the modulation of plant responses to AR stress [7]. Our results showed that the abundance of ICS (spot 4, Table 2) was up-regulated only in *T. wallichiana* var*. mairei*. Its presence may provide protection against AR stress and possibly endow *T. wallichiana* var*. mairei* with greater AR tolerance.

#### 2.2.6. Signal Transduction Related Proteins

In this study, three out of four signal transduction-related proteins, including putative signal tranduction protein (spot 25, Table 1), T-cell activation protein phosphatase 2C-like protein (spot 42, Table 1) and calcium-dependent protein kinase (spot 63, Table 1) showed a down-regulated expression in *P. massoniana* under simulated AR, whereas the abundance of light-mediated development protein DET1-like isoform 2 (spot 2, Table 2) was increased in *T. wallichiana* var*. mairei* under AR stress. Free cytosolic Ca^2+^ is a universal second messenger in plants, acting as a mediator of stimulus–response coupling in the regulation of growth, development and responses to environmental stresses [38]. Modulation of intracellular Ca^2+^ levels is partly regulated by calcium related proteins. In the present study, the expression of caleosin-related protein (spot 8, Table 1) was significantly down-regulated in *P. massoniana* under AR stress, suggesting that caleosin-related protein plays an important role in response to AR which is consistent with the previous results in *Arabidopsis* [39]. Previous studies showed that AR impacts on Ca nutrition, which causes alteration in membrane-associated Ca, membrane destabilization and foliar injury of red spruce, thus affecting forest health [40]. In this study, AR also led to the decreased abundance of a probable calcium-binding protein CML30 (spot 52, Table 1), calcium-binding protein CML19 (spot 8, Table 2) and calcium-binding protein KIC-like (spot 17, Table 2) in *P. massoniana* and *T. wallichiana* var*. mairei* under AR stress. This change indicates that Ca-binding proteins have a cryptic correlation with AR tolerance, and their roles need to be further analyzed in woody plants. Furthermore, calcium-dependent protein kinases (CDPKs) are implicated as major primary Ca^2+^ sensors in plants, and CDPK-controlled signaling pathways regulate specific responses to biotic and abiotic stresses. Here, the abundances of CDPK (spot 63, Table 1) was also decreased in AR-treated *P. massoniana*, suggesting that the modulation of Ca signaling regulators might reduce AR tolerance of *P. massoniana*. These findings indicate that Ca-dependent signal transduction could be an important signal pathway under AR stress in *P. massoniana* and *T. wallichiana* var*. mairei*.

#### 2.2.7. Transcription Related Proteins

Transcription is the first step in gene expression and a major point of regulation; transcriptional control on the expression of stress responsive genes is crucial for plant response to various abiotic and biotic stresses [28]. Five proteins including maturase K (spot 15), RNA polymerase beta subunit (spots 22, 23), transposase (spot 26) and retrotransposon protein (spot 56) in *P. massoniana* and three proteins including maturase K (spots 7, 26), RNA polymerase II C-terminal domain phosphatase-like 1 (spot 24) in *T. wallichiana* var*. mairei* were found to change their expression under AR stress (Tables 1 and 2). Maturase K catalyzes intron removal in RNA precursors and directly affects gene expression at the translation level. The DNA-dependent RNA polymerase (RNAP) is the central enzyme of the transcription cycle, and RNA polymerase beta subunit is one of the subunits composing the RNA polymerase catalytic core. In the current study, it is interesting that maturase K (spot 15, Table 1), RNA polymerase beta subunit (spots 22 and 23, Table 1) and auxin-responsive protein (spot 47, Table 1) were decreased in abundance in *P. massoniana* under AR treatment. However, the abundances of maturase K (spots 7 and 26, Table 2) and RNA polymerase II *C*-terminal domain phosphatase-like 1 (spot 24, Table 2) were increased in AR-treated *T. wallichiana* var*. mairei*, indicating that transcription related proteins play a critical role in response to AR stress through adjusting of basic genetic processes in plants. In our study, transcription related proteins showed decreases in abundance in AR-treated *P. massoniana*, whereas the abundance of transcription related proteins increased in AR-treated *T. wallichiana* var*. mairei* (Tables 1 and 2). These data suggest that the process of transcription may be different between *P. massoniana* and *T. wallichiana* var*. mairei* in response to AR.

In addition, transposase (spot 26, Table 1) and retrotransposon proteins (spot 56, Table 1) in transcription displayed down-regulated expression pattern in *P. massoniana* under AR stress. Further works on these proteins in woody plants under AR stress are needed to clarify their functions.

## 3. Experimental Section

### 3.1. Plant Materials and Experimental Procedure

The seedlings of *P. massoniana* and *T. wallichiana* var*. mairei* were grown in plastic pots containing 12 kg soil in a greenhouse with a light/dark regime of 16/8 h, temperature of 21/27 °C (night/day), relative humidity of 60%–70%, a light intensity of 390 μmol·m^−2^·s^−1^ photosynthetically active radiation (PAR). The seedlings were sprayed once each day with 200 mL·pot^−1^ distilled water as control (CK, pH 5.6) or simulated AR solution (AR, pH 3.0). The ion compositions of the CK solution were adopted from Liu *et al*., while AR solution was made from CK solution and the pH was adjusted with a mixture of H_2_SO_4_ and HNO_3_ in the ratio of 5 to 1 by chemical equivalents, which represents the average ion compositions of rainfall in southern China [10]. The final concentrations of SO_4_^2−^ and NO_3_^−^ were 0.45 and 0.09 mM, respectively. After 2-month simulated AR treatment, the sapling needles were collected for physiological measurements and proteomics research.

### 3.2. Chlorophyll Content and Net Photosynthetic Rate Measurements

Plant leaves (0.1 g of fresh weight (FW)) were prepared, and chlorophyll was extracted with ice-cold 80% *v*/*v* acetone. Absorption of the extract was measured at 663 and 646 nm with a spectrometer (Varian Cary 50 UV-VIS, Varian, Palo Alto, CA, USA) and the chlorophyll content was calculated as described by Wellburn [41].

Leaf net photosynthetic rate (*P*n) was measured with a portable photosynthesis system (Li-6400, Li-Cor, Lincoln, NE, USA). Air temperature, CO_2_ concentration, light intensity, and air relative humidity were maintained at 25 °C, 380 μ·L^−1^, 800 μmol·m^−2^·s^−1^ PAR, and 80%, respectively. At least ten saplings were randomly selected from the CK or AR treatment group for *P*n measurement.

### 3.3. Protein Extraction, 2-DE and 2-DE Gel Data Analysis

Total proteins were extracted from plant leaves according to the method of phenol extraction [42]. Final washed pellets were vacuum-dried and dissolved in lysis buffer (8 M urea, 2 M thiourea, 4% CHAPS, 1% DTT, 1% IPG buffer pH 4–7) at room temperature. Three independent biological repetitions were performed for each treatment. The protein concentration was determined with a 2-D Quant Kit (GE Healthcare Amersham Bioscience, Little Chalfont, UK) according to the manufacturer’s instructions.

Two-dimensional electrophoresis (2-DE) was performed according to Hu *et al*. [43]. The sample containing 500 μg proteins was loaded onto an IPG strip holder with 18-cm long, pH 4–7 linear (GE Healthcare, Piscataway, NJ, USA) IPG strip, and rehydrated for 18 h at room temperature. IEF was carried out using an Ettan IPGphor isoelectric focusing system (GE Healthcare Amersham Bioscience, Little Chalfont, UK) as follows: 300 V for 60 min, 600 V for 60 min, 1000 V for 60 min, a gradient to 8000 V for 120 min, and kept at 8000 V for a total of 64 000 Vh at 20 °C. After IEF, the IPG strips were equilibrated using an equilibration solution (6 M urea, 30% glycerol, 2% SDS, 50 mM Tris-HCl, pH 8.8) containing 1% DTT for 15 min, followed by 2.5% iodoacetamide in the same equilibration solution for 15 min. Electrophoresis in the second dimension was performed on 12.5% SDS polyacrylamide gels using a protean apparatus (Bio-Rad, Hercules, CA, USA) according to the manufacturer’s instructions. The gels were stained using Coomassie Brilliant Blue (CBB) (Bio-Rad, Hercules, CA, USA) R-250 and gel images were acquired at 600 dots per inch (dpi) resolution by a scanner (Uniscan M3600, Beijing, China). Three independent gels from each treatment were produced. 2-D gel analysis was performed with PDQuest software (version 7.0, Bio-Rad, Hercules, CA, USA) according to Liu *et al*. [15]. The protein spots that changed more than two-fold and passed the Student’s *t*-test with *p* < 0.05 were considered significant.

### 3.4. Protein Digestion and Identification

In-gel protein digestion and protein identification was followed as described by Liu *et al*. [15]. Matrix-assisted laser desorption/ionization-time-of-flight mass spectrometry (MALDI-TOF MS) analysis (ReFlexTM III, Bruker, Bremen, Germany) was used to acquire the peptide mass fingerprint (PMF). Standard peptide mixture was spotted adjacent to all samples for external calibration followed by internal mass correction using peptide ions generated by trypsin autoprotolysis (*m*/*z* 842.5, and *m*/*z* 2211.10). The spectra were analyzed with the flex analysis software (Version 3.2, Bruker-Daltonics, Bremen, Germany). Since the *P. massoniana* and *T. wallichiana* var*. mairei* genome are still unsequenced, a homology-based search was performed. The measured tryptic peptide masses were searched against the National Center for Biotechnology Information non-redundant (NCBInr, Bethesda, MD, USA) database (release date: 16 July 2012), and selecting the taxonomy of green plants using MASCOT interface (Version 2.0; Matrix Science, London, UK). The following parameters were used for database search: MH^+^ monoisotopic mass values, a fragment ion mass tolerance of ±0.3 Da, permitting one missed cleavage, alkylation of cysteine by carba-midomethylation as a fixed modification, and oxidation of methionine as a variable modification. MASCOT Peptide Mass Fingerprint (http://www.matrixscience.com) protein scores greater than 73 with the NCBInr database were considered significant (*p* < 0.05). A number of other criteria were further evaluated in the final assignment of protein and peptide identifications: the number of matching peptides (at least four), the coverage (a minimum of 9%), and the molecular weight (*M*r) and isoelectric point (p*I*) of the protein. Furthermore, to avoid false positives, an additional in-house Basic Local Alignment Search Tool (BLAST) search against the NCBI protein database (http://www.ncbi.nlm.nih.gov) was done to reconfirm all the matches.

The identified proteins were used to search for over/under-representation of the searched proteins adopting the AGI codes as input and then searched within the UniProt (Wellcome Trust Genome Campus, Cambridge, UK; Centre Medical Universitaire, Geneva, Switzerland; Georgetown University Medical Center, Washington, WA, USA, http://www.uniprot.org) and TAIR database (Carnegie Institution of Washington Department of Plant Biology 260, Stanford, CA, USA, http://www.arabidopsis.org) to find out if their functions are known, they were then further classified using Functional Catalogue software (Munich Information Center for Protein Sequences, Neuherberg, Germany, http://mips.gsf.de/projects/funcat).

### 3.5. Statistical Analysis

Values in figures and tables were expressed as means ± SE. The statistical significance of the data was analyzed using a univariate analysis of variance (*p* < 0.05) (Abacus Concepts, Berkeley, CA, USA, one-way ANOVA; SPSS for Windows, version 11.0, SPSS Inc., Chicago, IL, USA).

## 4. Conclusions

In this study, a comparative proteomics analysis was carried out to clarify the differentially expressed protein profiles of two tree species under AR treatment. Our data revealed that many more proteins altered their expression level in *P. massoniana* than in *T. wallichiana* var*. mairei* in response to simulated AR treatment. Taken together, metabolism, photosynthesis, signal transduction and transcription related proteins in *P. massoniana*, a sensitive species, were depressed by AR stress. However, the abundances of proteins participating in photosynthesis as well as in signal transduction and transcription were increased in *T. wallichiana* var*. mairei*, a tolerant species, under simulated AR. We presume that AR was a strongly oxidative situation experienced by *P. massoniana* but was of weak oxidative intensity in *T. wallichiana* var*. mairei*. On the other hand, AR stress influenced only part of the photosynthetic network and modified gene expression in such a way as to enhance metabolism systems and strengthen plant defense responses to maintain physiological and biochemical homeostasis in *T. wallichiana* var*. mairei*. This is likely one of the reasons *P. massoniana* is more sensitive to AR stress than *T. wallichiana* var*. mairei*. The proteins identified in this study might be useful in investigating the different defense mechanism of woody plants to AR stress.

**Figure 5.**
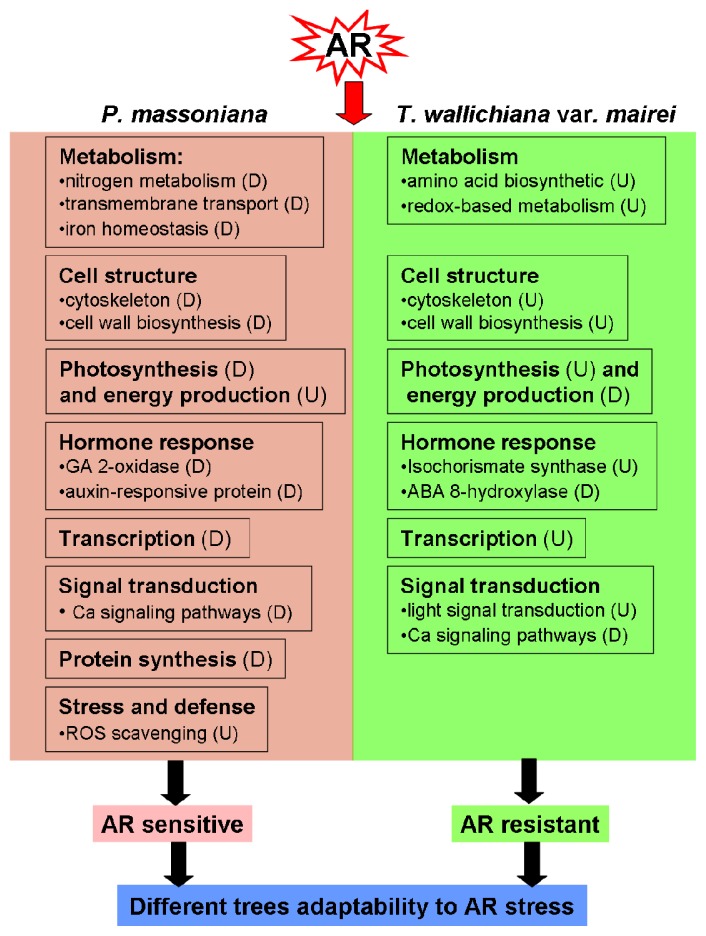
A schematic representation of different AR-tolerance mechanisms in *P. massoniana* and *T. wallichinana* var. *mairei* by regulating diverse biological processes. “U” or “D” indicate increase or decrease, respectively.

Based on the putative functions and expression changes of the identified proteins in *P. massoniana* and *T. wallichiana* var*. mairei*, together with previous reports, we outlined a schematic overview model associated with the different systematic response of *P. massoniana* and *T. wallichiana* var*. mairei* to AR stress (Figure 5). These results depict a panoramic view of the adaptation strategies in *P. massoniana* and *T. wallichiana* var*. mairei* under AR challenge and deepen our understanding in AR tolerance in woody plants.

## Supplementary Information



## Figures and Tables

**Table 1.** Identification of differentially expressed proteins using 2-DE and mass spectrometry analysis in *Pinus massoniana*.

Spot a	NCBI accession b	Protein identity c	Thero.kDa/p*I* d	Exper.kDa/p*I* e	SC f	MP/TP g	Score h	C i	Species
**Metabolism**									

4	gi|308807529	aminoimidazole ribonucleotide carboxylase	62.31/6.2	27.37/5.18	17%	9/14	97	U	*Ostreococcus tauri*
12	gi|90718161	granule bound starch synthase	37.38/7.11	27.42/5.44	31%	7/13	91	D	*Ficus hispidioides*
16	gi|357122042	UDP-arabinopyranose mutase 3-like	41.34/6.02	48.06/5.46	25%	9/12	95	U	*Brachypodium distachyon*
24	gi|224122152	mitochondrial phosphate carrier protein	38.64/9.18	44.04/5.56	21%	8/13	103	D	*Populus trichocarpa*
27	gi|435103	glyceraldehyde-phosphate dehydrogenase	44.66/7.55	51.45/6.65	32%	10/12	145	D	*Pinus sylvestris*
28	gi|396547	glutamate-ammonia ligase	39.79/6.42	52.00/6.55	20%	18/23	86	D	*Pinus sylvestris*
29	gi|308806413	putative D-protein	22.80/6.74	31.71/6.42	32%	7/9	98	D	*Ostreococcus tauri*
34	gi|357448955	nicotianamine synthase	32.03/8.11	35.56/5.88	25%	6/6	105	D	*Medicago truncatula*
39	gi|303281782	ABC transporter	130.84/6.55	85.07/5.89	10%	9/11	92	D	*Micromonas pusilla*
41	gi|13873338	lipoic acid synthase	41.75/8.61	84.86/5.95	23%	6/7	91	D	*Bruguiera gymnorhiza*
48	gi|190899164	2Fe-2S ferredoxin	18.33/8.52	20.94/6.21	38%	4/4	81	D	*Populus tremula*
54	gi|15240625	transaldolase	47.96/6.08	49.44/6.61	13%	6/6	92	D	*Arabidopsis thaliana*
57	gi|126583387	ferritin	28.26/5.66	49.03/5.29	24%	4/4	75	D	*Triticum urartu*

**Cell structure**									

62	gi|29028306	UDP-glucose dehydrogenase	53.52/6.06	87.31/4.70	21%	7/8	101	D	*Colocasia esculenta*

**Protein synthesis and modification**									

6	gi|357111489	oxygen-evolving enhancer protein 1	24.81/4.97	37.64/4.72	26%	6/10	91	U	*Brachypodium distachyon*
11	gi|46811008	small ribosomal protein 4	22.28/10.24	33.33/5.43	38%	6/9	87	U	*Haplomitrium blumei*
20	gi|308801835	Ulp1 protease family protein	68.17/9.21	22.11/6.90	22%	12/15	117	D	*Ostreococcus tauri*
21	gi|297830742	kinase family protein	43.77/5.79	33.72/5.55	31%	7/10	97	D	*Arabidopsis lyrata subsp*
31	gi|6525065	chloroplast translational elongation factor Tu	50.55/6.05	139.96/5.73	27%	9/11	109	D	*Oryza sativa Japonica* Group
37	gi|159477317	T-complex protein 1 beta subunit	57.40/5.37	58.03/5.88	30%	14/18	152	U	*Chlamydomonas reinhardtii*
45	gi|233142272	glycogen synthase kinase	47.06/8.64	31.97/6.00	25%	8/12	100	D	*Glycine max*
49	gi|255560267	chaperonin-60kD	61.48/6.20	24.54/6.25	14%	6/7	76	D	*Ricinus communis*

**Photosynthesis and energy production**									

3	gi|228016009	ATP synthase CF1 beta subunit	52.92/5.19	60.32/4.79	43%	18/30	161	U	*Pinus resinosa*
9	gi|357481701	Ycf2	108.70/8.98	32.16/6.10	13%	8/10	88	D	*Medicago truncatula*
17	gi|56784992	putative ATP synthase beta subunit	45.27/5.26	64.05/5.08	47%	13/19	167	U	*Oryza sativa Japonica* Group
18	gi|220938463	phosphoenolpyruvate carboxykinase (PEP carboxykinase)	55.85/6.63	89.72/5.19	19%	7/9	95	D	*Hyparrhenia hirta*
35	gi|18073888	phosphoenolpyruvate carboxylase(PPCK)	41.34/7.74	52.73/5.88	24%	6/7	94	D	*Leptotes bicolor*
44	gi|31281466	ribulose-1,5-bisphosphate carboxylase/oxygenase large subunit(Rubisco)	52.11/6.09	21.97/6.05	16%	8/14	88	D	*Pinus monophylla*
50	gi|166714465	ribulose-1,5-bisphosphate carboxylase/oxygenase large subunit(Rubisco)	51.60/6.00	59.41/6.11	36%	21/27	223	D	*Pinus parviflora*
53	gi|332591479	phosphoglycerate kinase 1	52.94/8.84	50.50/6.73	36%	15/22	153	D	*Pinus pinaster*
55	gi|34733684	ribulose-1,5-bisphosphate carboxylase/oxygenase large subunit(Rubisco)	48.97/6.34	21.49/4.63	14%	6/6	90	D	*Gnetum hainanense*
60	gi|264160443	ribulose 1,5-bisphosphate carboxylase	46.11/6.08	82.57/4.66	14%	8/11	87	D	*Callistephus chinensis*
65	gi|357111628	succinate dehydrogenase [ubiquinone] flavoprotein subunit	68.79/6.18	84.59/5.93	14%	7/8	90	U	*Brachypodium distachyon*

**Stress and defense**									

5	gi|255575353	peroxiredoxin	23.94/7.63	19.53/5.23	22%	5/6	83	D	*Ricinus communis*
10	gi|192912966	cytosolic ascorbate peroxidase	27.550/5.42	33.28/5.22	38%	7/9	98	U	*Elaeis guineensis*
13	gi|289187423	tau class glutathione S-transferase	26.47/5.56	29.44/5.44	36%	11/22	95	U	*Pinus brutia*
14	gi|289187423	tau class glutathione S-transferase	26.47/5.56	29.21/5.25	35%	10/14	103	U	*Pinus brutia*
30	gi|66841104	manganese superoxide dismutase	13.31/5.85	29.40/6.52	46%	5/6	97	D	*Larix gmelinii*
33	gi|154101561	phenylalanine ammonia-lyase	24.86/6.54	32.30/5.86	27%	5/7	84	D	*Scutellaria baicalensis*
36	gi|380863088	stromal ascorbate peroxidase	80.54/9.10	63.15/5.81	57%	4/4	92	D	*Dimocarpus longan*
43	gi|357513733	Tir-nbs-lrr resistance protein	140.72/5.31	140.40/5.95	12%	12/15	110	D	*Medicago truncatula*
51	gi|195620494	membrane-associated salt-inducible protein	43.66/9.02	114.61/6.19	20%	7/9	102	U	*Zea mays*
59	gi|308804281	cytochrome b5	16.34/7.90	55.92/5.86	45%	5/6	92	D	*Ostreococcus tauri*
64	gi|56481813	thiazole biosynthetic enzyme	36.63/6.01	35.00/5.51	29%	8/11	85	D	*Pseudotsuga menziesii*

**Hormone response**									

7	gi|212725010	abscisic acid and water-stress induced protein	16.30/9.55	40.39/4.95	28%	4/5	81	U	*Pinus sylvestris*
40	gi|224108798	gibberellin 2-oxidase	34.47/5.44	84.30/5.98	29%	8/10	106	D	*Populus trichocarpa*
47	gi|357485291	auxin-responsive protein	36.69/8.52	21.95/6.15	18%	6/6	80	D	*Medicago truncatula*

**Signal transduction**									

8	gi|145336050	caleosin-related protein	23.89/9.62	116.00/4.47	40%	6/7	97	D	*Arabidopsis thaliana*
25	gi|22128710	putative signal tranduction protein	97.72/6.16	54.98/5.62	13%	9/11	101	D	*Oryza sativa Japonica* Group
42	gi|226494574	T-cell activation protein phosphatase 2C-like protein	34.59/7.70	140.00/5.88	22%	5/5	84	D	*Zea mays*
52	gi|225425656	probable calcium-binding protein CML30	20.64/4.66	32.35/6.55	30%	5/5	97	D	*Vitis vinifera*
63	gi|357440111	calcium-dependent protein kinase	54.85/5.85	21.74/5.42	17%	7/8	84	D	*Medicago truncatula*

**Transcription**									

15	gi|384584973	maturase K	31.08/10.17	38.94/5.31	30%	6/8	100	D	*Cynodon nlemfuensis*
22	gi|313199657	RNA polymerase beta subunit	120.90/6.41	32.90/5.60	9%	8/9	78	D	*Isoetes flaccida*
23	gi|372482380	RNA polymerase beta subunit	158.24/9.38	38.65/5.46	11%	14/20	109	D	*Sansevieria trifasciata*
26	gi|18419497	transposase	15.77/8.93	69.37/5.69	44%	5/6	89	D	*Oryza nivara*
56	gi|108862655	retrotransposon protein	21.22/8.62	56.71/6.93	32%	6/8	93	D	*Oryza sativa Japonica* Group

**Function unknown and hypothetical proteins**									

1	gi|326501884	predicted protein	53.01/5.32	18.56/4.20	18%	7/11	84	D	*Hordeum vulgare subsp*
2	gi|21593511	pheromone receptor	39.28/4.44	33.13/4.56	24%	5/6	83	D	*Arabidopsis thaliana*
19	gi|118486611	unknown	43.94/6.43	35.36/6.07	19%	6/9	78	D	*Populus trichocarpa*
32	gi|116780007	unknown	25.59/5.82	30.20/5.92	45%	13/29	120	D	*Picea sitchensis*
38	gi|148906365	unknown	48.34/5.78	63.66/5.90	29%	11/16	109	U	*Picea sitchensis*
46	gi|116789937	unknown	54.01/6.00	57.72/6.04	23%	12/17	116	D	*Picea sitchensis*
58	gi|357488033	hypothetical protein MTR_5g047930	54.35/4.92	47.79/5.26	19%	6/9	78	D	*Medicago truncatula*
61	gi|242033729	hypothetical protein SORBIDRAFT_01g015060	18.87/4.75	36.24/4.67	32%	5/6	85	U	*Sorghum bicolor*

a Spot. is the unique differentially expressed protein spot number;

b Database accession numbers according to NCBInr;

c The name of the proteins identified by MALDI-TOF MS;

d Theoretical mass (kDa) and p*I* of identified proteins;

e Experimental mass (kDa) and p*I* of identified proteins;

f The amino acid sequence coverage for the identified proteins;

g Number of the matched peptides and the total searched peptides;

h The Mascot searched score against the database NCBInr;

i Spot abundance change. U stands for increased abundance of protein, D stands for decreased abundance of protein.

**Table 2.** Identification of differentially expressed proteins using 2-DE and mass spectrometry analysis in *Taxus wallichiana* var*.mairei*.

Spot a	NCBI accession b	Protein identity c	Thero.kDa/p*I* d	Exper.kDa/p*I* e	SC f	MP/TP g	Score h	C i	Species
**Metabolism**									

9	gi|3913651	ferredoxin-NADP reductase	40.71/8.37	11.75/5.40	35%	6/9	88	U	*Nicotiana tabacum*
13	gi|15228869	copper chaperone	13.08/4.91	39.62/5.55	33%	4/4	87	D	*Arabidopsis thaliana*
21	gi|334184891	aconitase/3-isopropylmalate dehydratase protein	23.88/6.16	28.02/4.47	36%	6/8	96	U	*Arabidopsis thaliana*

**Cell structure**									

14	gi|2500930	beta-fructofuranosidase	62.90/7.07	46.39/5.49	13%	6/6	94	U	*Pisum sativum*
23	gi|357521323	microtubule associated protein type 2	70.55/9.05	39.64/5.49	20%	9/12	102	D	*Medicago truncatula*
25	gi|159490038	eta tubulin	35.80/7.75	23.31/5.80	29%	6/8	94	U	*Chlamydomonas reinhardtii*

**Photosynthesis and energy production**									

1	gi|226498532	NADH-ubiquinone oxidoreductase 10.5 kDa subunit	11.28/9.15	15.08/4.64	47%	4/4	85	U	*Zea mays*
3	gi|138277483	ATP synthase beta subunit	51.67/5.11	63.61/4.90	42%	18/30	170	D	*Taxus brevifolia*
5	gi|138277483	ATP synthase beta subunit	51.67/5.11	62.00/5.08	45%	20/37	167	D	*Taxus brevifolia*
10	gi|357137138	phosphoribulokinase	45.31/5.97	40.14/5.21	27%	7/11	95	U	*Brachypodium distachyon*
11	gi|225459844	ATP-dependent zinc metalloprotease FTSH	75.76/6.36	84.83/5.33	27%	15/28	124	U	*Vitis vinifera*
12	gi|7592732	plasma membrane H^+^-ATPase	22.04/8.92	31.30/5.47	38%	6/6	102	U	*Nepenthes alata*
15	gi|150251443	ATP synthase CF1 alpha chain	55.36/5.38	57.82/5.53	15%	9/12	99	D	*Cycas taitungensis*
16	gi|154146830	phosphoenolpyruvate carboxylase	49.31/6.21	29.94/5.82	29%	8/10	118	U	*Cymbopogon citratus*
20	gi|350536787	chloroplast malate dehydrogenase	47.79/5.92	28.80/6.19	23%	8/8	110	U	*Solanum lycopersicum*

**Stress and defense**									

6	gi|357520455	cysteine proteinase inhibitor	25.78/7.78	28.19/5.04	21%	6/6	84	D	*Medicago truncatula*

**Hormone response**									

4	gi|76009223	isochorismate synthase	13.34/8.47	11.83/5.11	32%	4/4	82	U	*Solanum lycopersicum*
19	gi|335346406	abscisic acid 8-hydroxylase	53.18/8.77	30.53/6.17	17%	7/9	88	D	*Ipomoea nil*

**Signal transduction**									

2	gi|357132195	light-mediated development protein DET1-like isoform 2	48.61/8.53	49.88/4.76	17%	7/9	95	U	*Brachypodium distachyon*
8	gi|384245525	calcium-binding protein CML19 (centrin)	19.59/4.75	19.99/5.44	31%	4/4	78	D	*Coccomyxa subellipsoidea*
17	gi|356573251	calcium-binding protein KIC-like	14.00/4.18	28.30/5.78	47%	5/5	85	D	*Glycine max*

**Transcription**									

7	gi|154082680	maturase K	57.14/9.40	10.70/5.33	12%	6/6	85	U	*Haplophyllum buxbaumii*
24	gi|79481163	RNA polymerase II *C*-terminal domain phosphatase-like 1	109.15/5.81	30.99/5.62	15%	10/13	103	U	*Arabidopsis thaliana*
26	gi|15341050	maturase K	33.04/9.65	25.36/5.83	31%	7/11	99	U	*Persicaria runcinata*

**Function unknown and hypothetical proteins**									

18	gi|125547814	hypothetical protein OsI_15422	45.62/6.20	38.91/6.03	22%	7/10	94	U	*Oryza sativa Indica* Group
22	gi|296087931	unnamed protein product	79.30/7.32	15.63/5.96	20%	11/16	106	D	*Vitis vinifera*

a Spot. is the unique differentially expressed protein spot number;

b Database accession numbers according to NCBInr;

c The name of the proteins identified by MALDI-TOF MS;

d Theoretical mass (kDa) and p*I* of identified proteins;

e Experimental mass (kDa) and p*I* of identified proteins;

f The amino acid sequence coverage for the identified proteins;

g Number of the matched peptides and the total searched peptides;

h The Mascot searched score against the database NCBInr;

i Spot abundance change. U stands for increased abundance of protein, D stands for decreased abundance of protein.
