# Supplementary material for: Comparative Proteomic Analysis of Differential Responses of Pinus massoniana and Taxus wallichiana var. mairei to Simulated Acid Rain
Source: Int J Mol Sci. 2014 Mar 12;15(3):4333–55. doi: 10.3390/ijms15034333 (PMC3975401; doi:10.3390/ijms15034333)
Supplement: Supplementary file 1 [file ijms-15-04333-s001.pdf]

Supplementary Information

Table S1. The details of identified proteins in *Pinus massoniana* under AR stress.

Spot [a]	NCBI accession [b]	Protein identity [c]	Peptide sequence
		Metabolism	
4	gi|308807529	aminoimidazole ribonucleotide carboxylase	R.MLGIAASPMGVR.L + Oxidation (M)
			R.MLGIAASPMGVRLK.A + 2 Oxidation (M)
			K.RLAYDGR.G
			R.LAYDGR.G
			R.ARDGETR.A
			R.AILGWPLGDTSLKVGGAVMK.N + Oxidation (M)
			K.VGGAVMKNILGDAEGDEAMSR.A
			R.AHRLMGAALEVPGASIHWYEKPDMK.A
			K.MGHITVVGPSAAVATER.L
12	gi|90718161	granule bound starch synthase	K.IYGPIAGEDYQDNQLR.F
			R.FSLLCQAALEAPR.I
			R.FSLLCQAALEAPRILNFTSSK.Y
			K.YDASNVMSAKALLK.E
			R.NIPVIGFIGRLEEQK.G
			K.GSDILIAAIPHFIKENVQIIVLGTGK.K
			K.QLQQLEILYPGKAR.G

Table S1. *Cont.*

Spot [a]	NCBI accession [b]	Protein identity [c]	Peptide sequence
16	gi|357122042	UDP-arabinopyranose mutase 3-like	K.YIYTIDDDCFVAK.D
			K.NLLSPSTPFFFNTLYDPYR.D
			R.GYPFSLR.E
			K.GTLFPMCGMNLAFDR.E
			K.GTLFPMCGMNLAFDR.E + Oxidation (M)
			R.ELIGPAMYFGLMGDGQPIGR.Y
			R.ELIGPAMYFGLMGDGQPIGR.Y + Oxidation (M)
			K.TGLPYIWHSK.A
			K.ASDPFVNLKK.E
24	gi|224122152	mitochondrial phosphate carrier protein	K.YKSITSGFGVLLK.E
			R.GFFRGWVPTLLGYSAQGACK.F
			R.GWVPTLLGYSAQGACK.F
			R.QIPYTMMK.F + Oxidation (M)
			R.QIPYTMMKFASFETIVEQLYK.N
			K.FASFETIVEQLYK.N
			R.QLGLWGLFTR.G
			K.VFVGLPTTGGAAPA.-

Table S1. *Cont.*

**Spot** [a]	**NCBI accession** [b]	**Protein identity** [c]	**Peptide sequence**
27	gi|435103	glyceraldehyde-phosphate dehydrogenase	R.CWHGR.K
			K.DSPLDVVVINDTGGVK.Q
			K.YDSILGTFDPDVQVAGNDGISVDGK.V
			K.VVSDRNPVNLPWK.D
			K.GTMTTTHSYTGDQR.L
			R.VPTPNVSVVDLVVQVEK.K
			R.VPTPNVSVVDLVVQVEKK.T
			K.GILAVCDEPLVSIDFR.C
			K.VVAWYDNEWGYSQR.V
			R.VVDLADIVANNWK.S
28	gi|396547	glutamate-ammonia ligase	K.VIAEYIWIGGSGMDMR.S
			K.VIAEYIWIGGSGMDMR.S
			K.VIAEYIWIGGSGMDMR.S + Oxidation (M)
			K.VIAEYIWIGGSGMDMR.S + Oxidation (M)
			K.VIAEYIWIGGSGMDMR.S + 2 Oxidation (M)
			K.VIAEYIWIGGSGMDMR.S + 2 Oxidation (M)
			R.FIMER.I
			R.FIMER.I
			R.HKEHISAYGEGNER.R
			R.HKEHISAYGEGNER.R
			K.EHISAYGEGNER.R
			K.EHISAYGEGNER.R
			R.VGRDTEK.E
			R.VGRDTEK.E
			K.GYFEDR.R

Table S1. *Cont.*

Spot [a]	NCBI accession [b]	Protein identity [c]	Peptide sequence
			K.GYFEDR.R
			R.RPASNMDPYIVTSMIAETTILWKP.- + Oxidation (M)
			R.RPASNMDPYIVTSMIAETTILWKP.- + Oxidation (M)
29	gi|308806413	putative D-protein	R.ASSGASVTPNVTSQVRDR.F
			R.MYLADVWELDLR.R
			R.MYLADVWELDLRR.V
			R.RMVWTMLHDGVSPGMVPSGR.S + 2 Oxidation (M)
			R.MVWTMLHDGVSPGMVPSGR.S
			R.WTMIKENTASSMSGVGSAR.M + Oxidation (M)
			R.WTMIKENTASSMSGVGSAR.M + 2 Oxidation (M)
34	gi|357448955	nicotianamine synthase	K.LITLCGK.A
			K.NYLKLTHLEFTMFTK.H + Oxidation (M)
			R.MLFHTSDIVDVKNELK.E + Oxidation (M)
			K.EFNVVFLAALVGMDKK.E + Oxidation (M)
			K.VINHLAKYMAPGAILVLR.S + Oxidation (M)
			K.YMAPGAILVLR.S + Oxidation (M)
39	gi|303281782	ABC transporter	K.TWQAKCGALTVCGDLASR.V
			K.CGALTVCGDLASRVPAYFMR.N
			R.VLLQRTNFSLER.G
			K.EVGFDDAKMAATIQSLSGGWR.M
			K.MAATIQSLSGGWR.M
			K.NLTYYPMGFR.D + Oxidation (M)
			K.VIMYMKNVSFTYPGTTK.Q
			R.AALVGLNGAGKTTLMK.L

Table S1. *Cont.*

Spot [a]	NCBI accession [b]	Protein identity [c]	Peptide sequence
			K.LLIGELSPDEGVGEVWQHHNLR.L
41	gi|13873338	lipoic acid synthase	-.MQSRVPSLLAR.T
			R.LADESPSLGDFMNLQSGYSVEVGNK.K
			K.QSLEVLMAAR.E
			K.QSLEVLMAAREFSPPGTLTK.T
			R.EFSPPGTLTKTSIMLGCGETPDQVVK.S + Oxidation (M)
			R.AAGVDVITFGQYMRPSK.R + Oxidation (M)
48	gi|190899164	2Fe-2S ferredoxin	K.CGPMVLDALIK.I + Oxidation (M)
			R.EGICGSCAMNMDGCNGLACLTKIDK.S + 2 Oxidation
			K.IDKSGPPSMINPLPHMFVIK.D
			K.SIEPWLKR.K
54	gi|15240625	transaldolase	R.SFVNFRALNAK.L
			R.CSVSGGNGTAGK.R
			R.CSVSGGNGTAGKR.T
			K.RTTLHDLYEK.E
			K.VGEQLEDEGVDSFKK.S
			K.KSFESLLGTLQDK.A
57	gi|126583387	ferritin	-.MWPRVAPSPATAAAAAAVGQLSGAGLAAGSVR.L + Oxidation (M)
			R.LPGPLPSAAGSAVCCR.A
			K.FFKESSDEER.G
			K.ESSDEERGHADK.L

Table S1. *Cont.*

Spot [a]	NCBI accession [b]	Protein identity [c]	Peptide sequence
		Cell structure	
62	gi|29028306	UDP-glucose dehydrogenase	-.MVKICCIGAGYVGGPTMAVIALK.C<br>K.ILMHNSK.G<br>R.IITTNLWSAELSK.L<br>R.VVSSMFNTVSGKK.I<br>R.DLAMNKFDWDHPIHLQPMSPTAVK.Q + Oxidation (M)<br>R.VTWDAYEATK.G<br>K.GAHGVCILTEWDEFK.T
		Protein synthesis and modification	
6	gi|357111489	oxygen-evolving enhancer protein 1	K.AGKYEMK.K<br>K.FEEKDGIDYAAVTVQLPGGER.V<br>K.DGIDYAAVTVQLPGGER.V<br>R.VPFLFTVK.Q<br>R.GGSTGYDNAVALPAGGR.G<br>R.GGSTGYDNAVALPAGGRGDEEELAK.E
11	gi|46811008	small ribosomal protein 4	R.SGRTDQLIPNK.K<br>K.AKGSTGQVLLQLLEMR.L<br>R.LGMASTIPEAR.Q<br>R.LGMASTIPEARQLVNHR.H + Oxidation (M)<br>R.LGMAKGMDSIQSK.K + 2 Oxidation (M)<br>K.GVGLVNQIIDREWISLK.I
20	gi|308801835	Ulp1 protease family protein	R.HIALATRMVLSHR.S<br>R.ASGTLGAAPER.E<br>R.DEIDHLTPVLAPGGALDR.G

Table S1. *Cont.*

Spot [a]	NCBI accession [b]	Protein identity [c]	Peptide sequence
			K.TITDDGREAR.R
			R.ESAWRR.A
			K.RFGAVNIFGHALK.K
			K.SMISSFENGTMVGR.A + 2 Oxidation (M)
			R.QVLSSSTASALDEQLER.L
			R.VMESRSEGETQPR.A + Oxidation (M)
			R.SEGETQPRAHFFSTFFINK.L
			K.NGCDCGVFMIK.Y
			K.NGCDCGVFMIK.Y + Oxidation (M)
21	gi|297830742	kinase family protein	K.DCIDAKNNITTFDNISFK.T
			K.NNITTFDNISFKTDSSR.R
			R.VYKGHIETPEQVVAVK.Q
			R.ILVYEYMQNGSLEDHLLELAR.N + Oxidation (M)
			K.SDVYSFGVVFLEMITGR.R
			R.VIDTTKPTQEQNLVTWASPLFKDR.R
			K.TEEDGQTVEEEEEDERSK.L
31	gi|6525065	chloroplast translational elongation factor Tu	K.YDEIDAAPEER.A
			K.NMITGAAQMDGAILVVSGADGPMPQTK.E + 2 Oxidation (M)
			K.TMDDAMAGDNVGLLLR.G + Oxidation (M)
			K.DDIERGMVLAKPASITPHTK.F
			R.HSPFFPGYRPQFYMR.T
			R.VKMVVELIQPVACEQGMR.F + Oxidation (M)
			R.VKMVVELIQPVACEQGMR.F + 2 Oxidation (M)
			K.MVVELIQPVACEQGMRFAIPEGGK.T + 2 Oxidation (M)

Table S1. *Cont.*

Spot [a]	NCBI accession [b]	Protein identity [c]	Peptide sequence
37	gi|159477317	T-complex protein 1 beta subunit	K.VHPMTIIAGYR.E
			K.VHPMTIIAGYR.E + Oxidation (M)
			K.ILTVDKEHFANLAVDAIMR.L
			R.IENARILVANTPMDTDK.I + Oxidation (M)
			R.VDSMAKVAEIEAAEK.D
			K.VRSIIDHGINCFVNR.Q
			R.SIIDHGINCFVNR.Q
			R.LALVLGGEIVSTFDNPSEVK.L
			R.SLHDALCVLK.E
			R.VVYGGGWPEIR.M
			R.VVYGGGWPEIRMAK.A
			K.AVEELASR.T
			K.AAHGADSSSRMGVDVVR.G + Oxidation (M)
			R.GEAGDMKELGIYESFR.V + Oxidation (M)
45	gi|233142272	glycogen synthase kinase	R.EASGHGAAGVDRLPEEMNDMK.I + Oxidation (M)
			R.EMEATVVDGNGTETGHIIVTTIGGR.N
			K.QTISYMAER.V
			K.NRELQTMR.L
			R.MPLIYVKLYTYQIFR.A
			K.CMNPNYTEFK.F
			K.CMNPNYTEFKFPQIK.A + Oxidation (M)
			K.AHPWHKIFHK.R
49	gi|255560267	chaperonin-60kD	K.CELEDPLILIHEKK.I
			K.VCAIKAPGFGENR.K

Table S1. *Cont.*

Spot [a]	NCBI accession [b]	Protein identity [c]	Peptide sequence
			R.KASMQDLAVLTGGQVITEELGMNLEK.V + 2 Oxidation (M)
			K.VGAEAFGSCKK.V
			K.SGIIDPLK.V
			K.EPTAMAGGGGGMGY.- + 2 Oxidation (M)
		Photosynthesis and energy production	
3	gi|228016009	ATP synthase CF1 beta subunit	K.GQGTAGQEIQVTCEVQQLLGNHK.V
			R.AVAMSATDGLTR.G
			R.VIDTGAPLSVPVGGATLGR.I
			R.IFNVLGEPVDNLGPVDAR.I
			R.ITSPIHRPAPAFTELDTK.L
			K.LSIFETGIK.V
			K.VVDLLAPYR.R
			K.VVDLLAPYRR.G
			K.IGLFGGAGVGK.T
			K.AHGGVSVFGGVGER.T
			K.VALVYGQMNEPPGAR.M
			K.VALVYGQMNEPPGAR.M + Oxidation (M)
			R.VGLTALTMAEYFR.D
			R.VGLTALTMAEYFR.D + Oxidation (M)
			R.DVNEQDVLLFIDNIFR.F
			R.FVQAGSEVSALLGR.M
			R.MPSAVGYQPTLSTEMGSLQER.I
			R.MPSAVGYQPTLSTEMGSLQER.I + Oxidation (M)

Table S1. *Cont.*

Spot [a]	NCBI accession [b]	Protein identity [c]	Peptide sequence
9	gi|357481701	Ycf2	M.TSYFDLSTERSMNK.D
			R.NPLKPFHRSSLISSFLK.A
			K.LKSQMMYLFYNR.Y + Oxidation (M)
			R.GSPKFQQFVSIFHDIR.D
			R.HLSHTSIYGSSLYTLIR.K
			K.IESWILNSDFIADK.E
			K.YLATNSYVPFITVSLNK.F
			K.KIFDTNGCGSITLGSNVR.D
17	gi|56784992	putative ATP synthase beta subunit	-.MDGTEGLVRGQR.V + Oxidation (M)
			K.GDITTNHFLPIHR.E
			K.VVDLLAPYQR.G
			K.TVLIMELINNVAK.A
			K.AHGGFSVFAGVGER.T
			R.EGNDLYREMIESGVIK.L + Oxidation (M)
			K.CALVYGQMNEPPGAR.A
			R.VGLTGLTVAEHFR.D
			R.DAEGQDVLLFIDNIFR.F
			R.FTQANSEVSALLGR.I
			R.IPSAVGYQPTLATDLGGLQER.I
			R.QISELGIYPAVDPLDSTSR.M
			K.NLQDIIAILGMDELSEDDKLTVAR.A + Oxidation (M)
18	gi|220938463	phosphoenolpyruvate carboxykinase	K.GSFVTSTGALATLSGAK.T
			K.GSFVTSTGALATLSGAKTGR.S
			K.KGLFGLMHYLMPK.R + Oxidation (M)

Table S1. *Cont.*

Spot [a]	NCBI accession [b]	Protein identity [c]	Peptide sequence
			K.NVILLACDAFGVLPPVSK.L
			K.EPQATFSACFGAAFIMLHPTK.Y + Oxidation (M)
			K.TEVFGLEIPTEIK.G
			K.ETLLTLAGLFKK.N
35	gi|18073888	phosphoenolpyruvate carboxylase	R.GGGPTHLAILSQPPDTIHGSLR.V
			R.ALLDEMAVVATEEYRSIVFQEPR.F
			K.DIKNLHMLQEMYNAWPFFR.V + Oxidation (M)
			K.NLHMLQEMYNAWPFFR.V + 2 Oxidation (M)
			R.DLLEGDPYLKQR.L
			K.EIMDSNKTAAELVK.L + Oxidation (M)
44	gi|31281466	ribulose-1,5-bisphosphate carboxylase/oxygenase large subunit	R.NHGMHFR.V
			R.MSGGDHIHAGTVVGKLEGER.D
			R.DVTLGFVDLLR.D
			R.DVTLGFVDLLRDDFIEK.D
			R.VALEACVQAR.N
			K.WSPELAAACEIWK.E
			K.EIKFEFDVIDR.L
			K.FEFDVIDRL.-
50	gi|166714465	ribulose-1,5-bisphosphate carboxylase/oxygenase large subunit	R.LTYYTPEYQTK.D
			K.DTDILAAFR.V
			K.TFQGPPHGIQVER.D
			K.YGRPLLGCTIKPK.L

Table S1. *Cont.*

Spot [a]	NCBI accession [b]	Protein identity [c]	Peptide sequence
			R.AVYECLR.G
			R.GGLDFTKDDENVNSQPFMR.W
			R.GGLDFTKDDENVNSQPFMR.W + Oxidation (M)
			K.DDENVNSQPFMR.W
			K.DDENVNSQPFMR.W + Oxidation (M)
			R.ELGVPIVMHDYLTGGFTANTSLAHYCR.D
			R.ELGVPIVMHDYLTGGFTANTSLAHYCR.D + Oxidation
			R.DNGLLLHIHR.A
			R.QRNHGMHFR.V + Oxidation (M)
			R.NHGMHFR.V
			R.DVTLGFVDLLR.D
			R.DVTLGFVDLLRDDFIEK.D
			R.VALEACVQAR.N
			K.WSPELAAACEIWK.E
			K.EIKFEFDVIDR.L
			K.FEFDVIDR.L
			K.FEFDVIDRL.-
53	gi|332591479	phosphoglycerate kinase 1	R.ADLNVPLDENQNITDDTR.I
			K.YSLKPLVPR.L
			R.LTELLGVNVVK.A
			K.AVASLPNGGVLLLENVR.F
			K.KLASIADLYVNDAFGTAHR.A
			K.LASIADLYVNDAFGTAHR.A
			K.YLKPAVAGFLLQK.E

Table S1. *Cont.*

Spot [a]	NCBI accession [b]	Protein identity [c]	Peptide sequence
			K.IELAKTLMQK.A
			K.GVSLLLPTDVVIADK.F
			K.VVPASDIPDGWMGLDIGADSIK.T
			K.VVPASDIPDGWMGLDIGADSIK.T + Oxidation (M)
			K.TVIWNGPMGVFEFDK.F
			K.TVIWNGPMGVFEFDK.F + Oxidation (M)
			K.GVITIIGGGDSVAAVEK.V
			K.MSHISTGGGASLELLEGK.T
55	gi|34733684	ribulose-1,5-bisphosphate carboxylase/oxygenase large subunit	R.LTYYTPEYQTK.D
			K.DTDILAAFR.V
			K.TFQGPPHGIQVER.D
			K.YGRPLLGCTIK.H
			K.NHGMHFR.V + Oxidation (M)
			R.EITLGFVDLLR.D
60	gi|264160443	ribulose 1,5-bisphosphate carboxylase	-.EYETKDTDILAAFR.V
			K.DTDILAAFR.V
			R.DNGLLLHIHR.A
			R.QKNHGMHFR.V + Oxidation (M)
			K.NHGMHFR.V
			K.NHGMHFR.V + Oxidation (M)
			R.MSGGDHIHAGTVVGK.L
			R.EITLGFVDLLR.D

Table S1. *Cont.*

Spot [a]	NCBI accession [b]	Protein identity [c]	Peptide sequence
65	gi|357111628	succinate dehydrogenase [ubiquinone] flavoprotein subunit	R.AAIGLSEHGFNTACITK.L
			R.WHMYDTVK.G
			K.GSDWLGDQDSIQYMCR.E + Oxidation (M)
			K.AVIELENYGLPFSR.T
			R.LGANSLLDIVVFGR.A
			R.TQETLEEGCQLISK.A
			K.AWESFHDVK.I
		Stress and defense	
5	gi|255575353	peroxiredoxin	K.LSFSPLSLKLQSK.A
			K.HLPGFVEK.S
			K.GIDVIACVSVNDAFVMK.A + Oxidation (M)
			R.RYAILAEDGVVK.V
			R.YAILAEDGVVK.V
10	gi|192912966	cytosolic ascorbate peroxidase	R.IAWHSAGTYDVK.T
			K.GSDHLR.D
			R.DVFGHMGLSDQDIVALSGGHTLGR.C
			R.DVFGHMGLSDQDIVALSGGHTLGR.C + Oxidation (M)
			R.SGFEGAWTSNPLIFDNSYFK.E
			K.ELLSGEKEGLLQLPSDK.A
			K.YAADEDAFFADYAEAHLK.L
13	gi|289187423	tau class glutathione S-transferase	K.VLNLWASPFGLR.V
			K.GVKYEYQEENLASK.S

Table S1. *Cont.*

Spot [a]	NCBI accession [b]	Protein identity [c]	Peptide sequence
			R.FWADFVDKK.I
			K.KIYDNGGALIMK.C
			K.IPLETQFPR.L
			R.LHEWVNACMER.E
			R.LHEWVNACMER.E + Oxidation (M)
			K.VLPHPEKVAEFAMQMR.Q
			K.VAEFAMQMR.Q
			K.VAEFAMQMR.Q + Oxidation (M)
			K.VAEFAMQMRQR.F + Oxidation (M)
14	gi|289187423	tau class glutathione S-transferase	K.VLNLWASPFGLR.V
			K.GVKYEYQEENLASK.S
			R.FWADFVDKK.I
			K.IYDNGGALIMK.C + Oxidation (M)
			K.IPLETQFPR.L
			R.LHEWVNACMER.E
			K.VLPHPEKVAEFAMQMR.Q
			K.VAEFAMQMR.Q
			K.VAEFAMQMR.Q + Oxidation (M)
			K.VAEFAMQMR.Q + 2 Oxidation (M)
30	gi|66841104	manganese superoxide dismutase	K.AIDTQFGSLNAVIEK.M
			K.GLLPLLGIDVWEHAYYLQYK.N
			K.NVRPDYLK.N
			K.YAEEIFEK.E
			K.YAEEIFEKEINP.-

Table S1. *Cont.*

Spot [a]	NCBI accession [b]	Protein identity [c]	Peptide sequence
33	gi|154101561	phenylalanine ammonia-lyase	-.DWVMESMNK.G + Oxidation (M)
			-.DWVMESMNKGTDSYGVTTGFGATSHR.R + 2 Oxidation (M)
			K.GTDSYGVTTGFGATSHRR.T
			R.AAMLVRINTLLQGYSGIR.F
			K.HHPGQIEAAAIMEHILDG.-
36	gi|380863088	stromal ascorbate peroxidase	K.CAASDPVQLKSAR.E
			K.TKFCHPILVR.L
			K.FCHPILVR.L
			R.LGWHDAGTYNKNIEEWPQR.G
43	gi|357513733	Tir-nbs-lrr resistance protein	K.AIDDSHMSLVVFSK.D + Oxidation (M)
			K.AIDDSHMSLVVFSKDYATSK.W + Oxidation (M)
			K.DYATSK.W
			K.LNGHVVIPVFYNIDPSHVRHQK.E
			K.SHVDKVSEWK.A
			K.LALMYPNELKDIVK.V
			K.DIVKVDENSEHIELLLK.T
			K.VDEIYEVKTWK.L
			K.QEFWESELNLYENK.G
			K.DLELELYQEIQFER.S
			K.ENLDDDDDDNSKQMVK.S + Oxidation (M)
			K.SKILYENCTMSGEETETSSHK.Q
51	gi|195620494	membrane-associated salt-inducible protein	-.MAAAVRHIVR.R + Oxidation (M)
			R.SFYSVFDEMCKK.G
			K.KGLKPNSITFTTALAGFYK.E

Table S1. *Cont.*

**Spot** [a]	**NCBI accession** [b]	**Protein identity** [c]	**Peptide sequence**
			K.GLKPNSITFTTALAGFYKEEK.F
			R.SGEARALVDEMMK.K + Oxidation (M)
			K.GTKPNWVTYNHLIYGFCK.E
			K.GTKPNWVTYNHLIYGFCKEGDLEEAK.R
59	gi|308804281	cytochrome b5	M.DDPSRPR.S
			M.DDPSRPRSAPASAGTHAKPSTTSQSVGK.I
			K.ITLRPGYSQMDWLR.R + Oxidation (M)
			R.SFGTDATALFDKYHK.W
			K.YHKWVNGEYIMR.A
64	gi|56481813	thiazole biosynthetic enzyme	K.YDLQSFK.F
			R.VSGVVTNWALVSMNHDTQSCMDPNVMESK.V + 2 Oxidation (M)
			R.VSGVVTNWALVSMNHDTQSCMDPNVMESK.V + 3 Oxidation (M)
			K.VVVSSCGHDGPFGATGVK.R
			K.SVGMIDKVPGMK.A + Oxidation (M)
			R.EIVPGMIVTGMEVAEIDGSPR.M
			R.EIVPGMIVTGMEVAEIDGSPR.M + Oxidation (M)
			R.MGPTFGAMMVSGQK.A + Oxidation (M)
		Hormone response	
7	gi|212725010	abscisic acid and water-stress induced protein	K.ERFGELGTVAAGGYALYER.N
			R.FGELGTVAAGGYALYER.N
			R.HKTEEEVAAAGAVGTGGYAFHER.H
			K.TEEEVAAAGAVGTGGYAFHER.H

Table S1. *Cont.*

Spot [a]	NCBI accession [b]	Protein identity [c]	Peptide sequence
40	gi|224108798	gibberellin 2-oxidase	K.MENESFNFFAK.T
			K.QKAGLDNSFGYGCK.N
			R.ELACELLDLMAEGLWVPDR.S
			R.LNHYPAMPILCK.D + Oxidation (M)
			K.SRMSMAYFAAPPLNAR.I
			K.SRMSMAYFAAPPLNAR.I + Oxidation (M)
			R.LGDSRLGLFMLEVDDQVA.- + Oxidation (M)
			R.LGLFMLEVDDQVA.- + Oxidation (M)
47	gi|357485291	auxin-responsive protein	K.NVVSGNKR.G
			K.NSMATASK.N
			K.NSMATASK.N + Oxidation (M)
			K.NSMATASKNNNDEVDGKPGPAALFVK.V + Oxidation (M)
			K.EMMSESK.L
			K.DGDWMLVGDVPWEMFIDTCR.R
		Signal transduction	
8	gi|145336050	caleosin-related protein	-.MSHQTVALASKAK.S + Oxidation (M)
			K.MTALEKHVSFFDR.N
			K.DGTVYPWETYQGFR.A
			R.LLAAFVAIFINMGLSK.K + Oxidation (M)
			K.DALTAEEIQKMLK.T
			K.SVRAIYDGSLFHQLEK.K
25	gi|22128710	putative signal tranduction protein	R.FPISSDRAHVHSLAFTWHDAFK.T
			R.AHVHSLAFTWHDAFKTGK.K

Table S1. *Cont.*

Spot [a]	NCBI accession [b]	Protein identity [c]	Peptide sequence
			R.GVAAPLLDSVNKLESNMK.T
			K.LESNMKTNLER.A
			R.VPDASSLGALPAASLVKPTSLAEVLDASK.E
			K.ERLFSSLVPDGSMK.A + Oxidation (M)
			R.LFSSLVPDGSMKALSK.Y
			K.QQCSDFIMTRNIQCR.E
			R.EMIEDVQKK.L + Oxidation (M)
42	gi|226494574	T-cell activation protein phosphatase 2C-like protein	R.FVASVLGDWMETEYATSER.K + Oxidation (M)
			K.VGSMPAAR.D + Oxidation (M)
			R.MGTNLGFSPKNMADIIAGIAYGISK.D
			K.WACTPFGMGYMKVHGLAR.R
			K.VHGLARR.G
52	gi|225425656	probable calcium-binding protein CML30	K.SPDPDPVLGPQHDTGHNR.K
			K.SPDPDPVLGPQHDTGHNRK.L
			K.GGELKMVMDR.L + Oxidation (M)
			R.VLCSLGLKEGSQVEDCR.R
			K.EFVKFLDK.S
63	gi|357440111	calcium-dependent protein kinase	K.VASMTETILNAK.Q
			R.TTGEVLACKSIAK.D
			R.WFLYCHENGIVHR.D
			R.FPSEPWDRISK.S
			K.IESSGEWHVGGGSFSASFMSR.N
			K.IESSGEWHVGGGSFSASFMSR.N + Oxidation (M)

Table S1. *Cont.*

**Spot** [a]	**NCBI accession** [b]	**Protein identity** [c]	**Peptide sequence**
			R.WLSCTQLPTSPKSSLVC.-
		Transcription	
15	gi|384584973	maturase K	-.PSLHLLRFFLNYYSNWNSLITSMK.S + Oxidation (M)
			R.FFLNYYSNWNSLITSMK.S + Oxidation (M)
			R.LSRFLYNSYVSEYEFFLLFLR.K
			R.IRLNQLTNSCFDFLGYR.S
			K.KFDTTAPGTPLIGSLAK.A
			K.FDTTAPGTPLIGSLAK.A
22	gi|313199657	RNA polymerase beta subunit	K.AKHIQSR.E
			R.EYAISELYKLLYGTDEYLK.F
			R.MNRATQHK.R
			K.CIVGTGLEGQVAPDSGTVVVAAR.G
			R.MNVGQIFECVLGIAGDFLR.R
			R.MNVGQIFECVLGIAGDFLR.R + Oxidation (M)
			R.MNVGQIFECVLGIAGDFLRR.H
			R.QKVVSTIVAGEPIYEPEVITPESFR.L
23	gi|372482380	RNA polymerase beta subunit	K.VDRFFFIPEEVHILPGSSSIMVR.N + Oxidation (M)
			K.NGSMEEVHASFVEVR.A + Oxidation (M)
			K.NGSMEEVHASFVEVRANDLIR.D + Oxidation (M)
			K.IPLVTQHQGTIGTLLNRNK.E
			K.YNNVVKESNPITPIR.G
			K.QTFQVLQVLKYCLIDENK.R
			K.YCLIDENKR.I
			K.YGPHIKK.S

Table S1. *Cont.*

Spot [a]	NCBI accession [b]	Protein identity [c]	Peptide sequence
			R.RVEGWNER.I
			R.HIEIIVWQVTSK.V
			R.AIFLGITR.A
			R.GRIDWLK.G
			K.ENVVLGGIIPVGTGFKK.L
			R.QDKNIHLEIK.K
26	gi|18419497	transposase	K.SEKYYLLTEEDDPHR.T
			K.YYLLTEEDDPHRTFK.N
			R.FMFLCVCARPRFR.D + Oxidation (M)
			R.GDLVMKPITSITR.D
			R.EDVGKPIFIQQDNA.-
56	gi|108862655	retrotransposon protein	R.GAWEYTGYNDPMRTHVGER.W
			R.THVGER.W
			R.DGQELADPGLR.S
			R.SLHTPQRGPR.A
			R.QIPTFPESPNLQEK.T
			K.QPFEHVDPV.-
Function unknown and hypothetical proteins			
1	gi|326501884	predicted protein	K.FLSKLLALSPGSMGK.F
			K.LLALSPGSMGK.F
			K.TVASMETETVVRK.S + Oxidation (M)
			R.VYSLTLPDPPIGDR.Y
			R.YLIGSSHGWLITADDK.S
			R.YNPDLNPQMVGPK.I + Oxidation (M)
			K.TNPRDIGVLNLEDDSR.E

Table S1. *Cont.*

Spot [a]	NCBI accession [b]	Protein identity [c]	Peptide sequence
2	gi|21593511	pheromone receptor	R.SIAADSWSIKSEYGSTLDDDQR.H
			R.HADAAEALSSANFR.V
			R.HADAAEALSSANFRVSSDYSSDK.E
			K.LVMDKGTLDAIGLHPDGPVK.R
			R.TYPTFMFGGSVGSRVATVAFLR.K + Oxidation (M)
19	gi|118486611	unknown	K.GMALQQRK.S + Oxidation (M)
			K.KLSEEEQNTLR.A
			R.AASLLQDLAKEKPGDPDVFR.L
			K.NVDFEVLRGHANALLAAK.K
			R.GYLAKGIILK.E
			K.GIILKENGNVGDAER.M
32	gi|116780007	unknown	K.LVQIEHALMAVGSGQTSLGIK.A
			K.LVQIEHALMAVGSGQTSLGIK.A + Oxidation (M)
			K.KLPSILVDESSVQK.I
			K.IQLLTSNIGVTYSGMGPDSR.V
			K.IQLLTSNIGVTYSGMGPDSR.V + Oxidation (M)
			R.KQAQQYYK.L
			K.LYKEPIPVTQLVR.E
			K.EPIPVTQLVR.E
			K.RYTEDMELDDAVHTAILTLK.E
			R.YTEDMELDDAVHTAILTLK.E
			R.YTEDMELDDAVHTAILTLK.E + Oxidation (M)
			K.NIEIGVVGTDR.K
			K.NIEIGVVGTDRK.F

Table S1. *Cont.*

Spot [a]	NCBI accession [b]	Protein identity [c]	Peptide sequence
38	gi|148906365	unknown	R.AAVPSGASTGVYEALELR.D
			K.KIPLYQHIANIAGNK.T
			K.LAMQEFMILPVGASSFR.E
			K.LAMQEFMILPVGASSFR.E + Oxidation (M)
			K.LAMQEFMILPVGASSFR.E + 2 Oxidation (M)
			R.EAMQMGSEVYHHLK.A
			K.YGQDATNVGDEGGFAPNIQENK.E
			K.VVIGMDVAASEFYTEK.D
			K.YDLNFKEENNNGSQK.I
			R.AGWGVMTSHR.S
			K.YNQLLR.I
46	gi|116789937	unknown	M.APRPLDYECINENVK.K
			R.GELYLR.A
			K.IIFTNVGNPQALGQRPLTFPR.Q
			K.HYLAMTTGGLGAYSDSR.G
			K.EVAEFIER.R
			K.GVMQILNTIIR.D
			K.GVMQILNTIIR.D + Oxidation (M)
			K.GVMQILNTIIRDEK.D
			K.GVMQILNTIIRDEK.D + Oxidation (M)
			R.AMVIINPGNPTGQCLSR.D
			K.GYWGECGQR.G
			K.EGVFHLR.T

Table S1. *Cont.*

Spot [a]	NCBI accession [b]	Protein identity [c]	Peptide sequence
58	gi|357488033	hypothetical protein MTR_5g047930	M.GVELDSQSFEFNDSDENSPTFK.S<br>K.KIEHGFDSSPNDMK.S<br>K.IEHGFDSSPNDMKSANCMPDPK.L<br>R.SSNDVGSFMMFATDK.Q<br>K.AMDLHEDMHDSVAPDDK.D<br>R.RGFGESSFSAAGAVSGR.I
61	gi|242033729	hypothetical protein SORBIDRAFT_01g015060	R.SFGFVTFLER.E<br>R.QQQEEEMQR.L<br>R.AAMQAAEKLHR.E<br>R.EKLAAER.E<br>K.EEETADPMAAAEAQAVK.Q

[a] Assigned spot number as indicated in Figure 2A and Table 1; [b] Database accession numbers according to NCBInr; [c] The name of the proteins identified by MALDI-TOF MS.

Table S2. The details of identified proteins in *Taxus wallichiana* var. *mairei* under AR stress.

Spot [a]	NCBI accession [b]	Protein identity [c]	Peptide sequence
		Metabolism	
9	gi|3913651	ferredoxin-NADP reductase	-.MATAVSAAVSLPSSKSTSFSSR.T + Oxidation (M)<br>K.INFNKVPLYYR.N<br>K.EMLMPKDPNATVIMLATGTGIAPFR.S + 2 Oxidation (M)<br>K.HEDYKFNGTAWLFLGVPTSSSLLYK.E<br>K.MYIQTRMAQYAEELWTLLQK.D + Oxidation (M)<br>K.GMEQGIDEIMSALAERDGIVWADYK.K
13	gi|15228869	copper chaperone	-.MAQTVVLKVGMSCQGCVGAVNR.V + 2 Oxidation (M)<br>K.VGMSCQGCVGAVNRVLGK.M<br>R.VLGKMEGVESFDIDIK.E + Oxidation (M)<br>K.MEGVESFDIDIKEQK.V + Oxidation (M)
21	gi|334184891	aconitase/3-isopropylmalate dehydratase protein	-.MAASLQSANPTLSR.T<br>R.TLASPNKPSSFATFR.S<br>K.LGSYALVGLPASYK.E<br>K.AVVAQSYARIFFR.N<br>R.VCDECTTGDVATVELR.E<br>K.AGMIPSAAA.- + Oxidation (M)
		Cell structure	
14	gi|2500930	beta-fructofuranosidase	K.SPKNPLMEPTIANK.I + Oxidation (M)<br>K.NPLMEPTIANK.I<br>K.HPLHSAEGTGMWECPDFYPVLDK.N

Table S2. *Cont.*

**Spot** [a]	**NCBI accession** [b]	**Protein identity** [c]	**Peptide sequence**
			K.HPLHSAEGTGMWECPDFYPVLDKNLLR.T
			K.GAGVKGGVGPFGLLVFASQGLK.E
			R.VYPTLAIHDK.A
23	gi|357521323	microtubule associated protein type 2	-.MAEFSGDVGMEPTVVTPVPLTVSGSFK.E
			R.ELSEAQAEIKALK.H
			K.DDDMPPIEAILAPLEAELK.L
			R.MHRLK.V
			R.ELEEAVLAGGAAANAVRDYQR.K
			K.DANDKVMPVK.Q + Oxidation (M)
			R.GTSEGKCVSNGPSR.R
			K.ILLNGKPPSSSFNQSSER.T
			K.ILLNGKPPSSSFNQSSERTK.D
25	gi|159490038	eta tubulin	M.LIHSLAGGSGSGLGSRLLEHLR.Q
			R.QEFPLAHLAAASVTPR.L
			R.ADALRGNAPISTAGALLMAR.G
			R.GNAPISTAGALLMAR.G
			R.GYPIAAVPPGDGGPAQAALLSAYVK.S
			K.SLGPSGAAPTLAPEASLWLR.S
	Photosynthesis and energy production		
1	gi|226498532	NADH-ubiquinone oxidoreductase 10.5 kDa subunit	R.VLFCQSSPASAPAREFVK.K
			K.KNYGDIK.A
			R.YDMGVERCVNLDGLTEAQIDK.K
			R.CVNLDGLTEAQIDKK.L

Table S2. *Cont.*

Spot [a]	NCBI accession [b]	Protein identity [c]	Peptide sequence
3	gi|138277483	ATP synthase beta subunit	R.IAQIIGPVLDVSFPPGNMPK.I
			R.GMIVIDTGAPLSVPVGETTLGR.I
			R.GMIVIDTGAPLSVPVGETTLGR.I + Oxidation (M)
			R.IFNVLGEPVDDLGPVNALTTSPIHR.S
			K.FSIFETGIK.V
			K.VVDLLAPYR.R
			K.AHGGVSVFGGVGER.T
			K.VALVYGQMNEPPGAR.M
			K.VALVYGQMNEPPGAR.M + Oxidation (M)
			R.VGLTALTMAEYFR.D
			R.VGLTALTMAEYFR.D + Oxidation (M)
			R.DVNKQDVLLFIDNIFR.F
			K.QDVLLFIDNIFR.F
			R.FVQAGSEVSALLGR.M
			R.MPSAVGYQPTLSTEMGSLQER.I
			R.YKELQDIIAILGLDELSEEDR.L
			K.ELQDIIAILGLDELSEEDR.L
			K.ELQDIIAILGLDELSEEDRLTVAR.A
5	gi|138277483	ATP synthase beta subunit	R.IAQIIGPVLDVSFPPGNMPK.I
			R.AVAMSATDGLMR.G
			R.GMIVIDTGAPLSVPVGETTLGR.I
			R.GMIVIDTGAPLSVPVGETTLGR.I + Oxidation (M)
			R.IFNVLGEPVDDLGPVNALTTSPIHR.S

Table S2. *Cont.*

Spot [a]	NCBI accession [b]	Protein identity [c]	Peptide sequence
			K.FSIFETGIK.V
			K.VVDLLAPYR.R
			K.AHGGVSVFGGVGER.T
			K.VALVYGQMNEPPGAR.M
			K.VALVYGQMNEPPGAR.M + Oxidation (M)
			R.VGLTALTMAEYFR.D
			R.VGLTALTMAEYFR.D + Oxidation (M)
			R.DVNKQDVLLFIDNIFR.F
			K.QDVLLFIDNIFR.F
			R.FVQAGSEVSALLGR.M
			R.MPSAVGYQPTLSTEMGSLQER.I
			R.MPSAVGYQPTLSTEMGSLQER.I + Oxidation (M)
			R.YKELQDIIAILGLDELSEEDR.L
			K.ELQDIIAILGLDELSEEDR.L
			K.ELQDIIAILGLDELSEEDRLTVAR.A
10	gi|357137138	phosphoribulokinase	-.MAICSAHTTTSLRSPCTTISNTGLR.Q
			R.RLTSVFGGAAEPPK.G
			K.GGNPDSNTLISDTTTVICLDDYHSLDR.T
			R.VRDLLDFSIYLDISNEVK.F
			R.DLLDFSIYLDISNEVK.F
			R.KPDFDAYIDPQK.Q
			R.LDELIYVESHLSNLSTK.F
11	gi|225459844	ATP-dependent zinc metalloprotease FTSH	R.RAQGGPGGPGGLGGPMDFGR.S
			R.AQGGPGGPGGLGGPMDFGR.S

Table S2. *Cont.*

Spot [a]	NCBI accession [b]	Protein identity [c]	Peptide sequence
			R.AQGGPGGPGGLGGPMDFGR.S + Oxidation (M)
			K.LELQEVVDFLK.N
			R.AVAGEAGVPFFSCAASEFVELFVGVGASR.V
			K.SKAPCIVFIDEIDAVGR.Q
			K.APCIVFIDEIDAVGR.Q
			R.TPGFTGADLQNLMNEAAILAAR.R
			K.LVAYHEAGHALVGALMPEYDPVAK.I
			R.GQAGGLTFFAPSEER.L
			R.LESGLYSRSYLENQMAVALGGR.V + Oxidation (M)
			R.SYLENQMAVALGGR.V
			R.SYLENQMAVALGGR.V + Oxidation (M)
			K.DYSMATADIVDAEVR.E + Oxidation (M)
			K.ETVDGEEFMSLFIDGK.A
12	gi|7592732	plasma membrane H^+-ATPase	-.TGTLTLNKLTVDK.N
			K.GIDANTVVLMAAR.A
			R.TALTYIDGQGQMHR.V + Oxidation (M)
			K.GAPEQILNMAHNK.S + Oxidation (M)
			R.VHAVIDKFAER.G
			R.SLAVAYQEVTEKR.M
15	gi|150251443	ATP synthase CF1 alpha chain	R.VVNALAQPIDGR.G
			R.RSVYEPLQTGLIAIDSMIPIGR.G
			R.SVYEPLQTGLIAIDSMIPIGR.G
			R.SVYEPLQTGLIAIDSMIPIGR.G + Oxidation
			K.GQDVICVYVAIGQK.A

Table S2. *Cont.*

Spot [a]	NCBI accession [b]	Protein identity [c]	Peptide sequence
			K.EQHTLIIYDDLSK.Q
			R.KFLVQLR.E
			K.FLVQLR.E
			K.EAIQEHIELFLLR.E
16	gi|154146830	phosphoenolpyruvate carboxylase	-.HTDVIDAITTHLGIGSYR.S
			K.GKRPLLPPDLPMTEEIADVIGAMK.V
			K.LFSTDWYINHIGGK.Q
			K.QQVMVGYSDSGKDAGR.L
			R.LSAAWQLYVAQEEMAK.V + Oxidation (M)
			R.FTAATLEHGMHPPVSPKPEWR.K + Oxidation (M)
			R.FTAATLEHGMHPPVSPKPEWRK.L
			K.LMEEMAVVATEEYRSVVVK.E + 2 Oxidation (M)
20	gi|350536787	chloroplast malate dehydrogenase	-.MEEAEFIPSSSLTK.T
			K.KTECFGVFCLTYDLK.D
			K.KLINVSVSGAAGMIANHFLFK.L
			K.KLINVSVSGAAGMIANHFLFK.L + Oxidation (M)
			R.SGLLDINVQIFAEQGK.A
			R.GGVLIQK.W
			K.WGRSSAASTAVSVVDAMR.S
			K.DIVFSMPCRSK.G
		Stress and defense	
6	gi|357520455	cysteine proteinase inhibitor	K.DMFCCRPREVK.W
			K.DMFCCRPREVK.W + Oxidation (M)

Table S2. *Cont.*

Spot [a]	NCBI accession [b]	Protein identity [c]	Peptide sequence
			R.EVKWSR.Y
			K.FVNVVNAEFRR.C
			R.RCGAMGYHITLK.A
			K.YGRLHWFLSYV.-
		Hormone response	
4	gi|76009223	isochorismate synthase	R.LSICSEALAGTR.A
			R.RLEAVCSSVIIEPK.K
			R.LEAVCSSVIIEPKK.A
			R.GRLQAEDDEFK.I
19	gi|335346406	abscisic acid 8-hydroxylase	-.MEFIMVMLFTLATIFFLK.K + 3 Oxidation (M)
			-.MEFIMVMLFTLATIFFLKK.W + Oxidation (M)
			K.YGSIFK.T
			K.THILGCPCVMISSPEAAKVVLVTK.A + Oxidation (M)
			K.ERMLGK.Q
			K.LVLRAFMPDALK.N
			K.GKEGQVLCWEHTK.K
		Signal transduction	
2	gi|357132195	light-mediated development protein DET1-like isoform 2	K.QRLLSFIFR.K
			R.LLSFIFR.K
			R.LLSFIFRK.T
			K.TWNEEPDQTLRVQHLK.K

Table S2. *Cont.*

Spot [a]	NCBI accession [b]	Protein identity [c]	Peptide sequence
			K.MMASLPYTCQSQSPSPYFDLTLFR.Y
			R.QPNVVK.F
			K.IKPGSDSGASDSRAK.R
8	gi|384245525	calcium-binding protein CML19 (centrin)	K.VAMRALGFEPK.K + Oxidation (M)
			K.DGSGTIDFDEFLTMMTAK.M + 2 Oxidation (M)
			K.DGSGTIDFDEFLTMMTAKMDER.D + 3 Oxidation (M)
			K.ELGENMTDEELQEMIDEADR.D
17	gi|356573251	calcium-binding protein KIC-like	K.EITVEVEEFEDLLPVMAKK.L
			R.NSTLLGMDGMSKEEAETMVR.Q + Oxidation (M)
			R.NSTLLGMDGMSKEEAETMVR.Q + 2 Oxidation (M)
			K.EEAETMVRQGDLDGDGK.L + Oxidation (M)
			R.QGDLDGDGKLNETEFCILMVR.L + Oxidation (M)
		Transcription	
7	gi|154082680	maturase K	R.MPLESGFYDNK.S + Oxidation (M)
			R.TYFYRK.V
			K.DPNVHYVRYQGK.L
			R.NLSHYYSGSSK.K
			R.LSCVKSLAR.K
			R.VSSTSRSFYLYR.G
24	gi|79481163	RNA polymerase II *C*-terminal domain phosphatase-like 1	R.DNKTAVMLLGGEELHLVAMYSENIK.N + 2 Oxidation (M)
			R.CLGIVFDLDETLVVANTMR.S
			R.LAVIVAEMKR.Y

Table S2. *Cont.*

Spot [a]	NCBI accession [b]	Protein identity [c]	Peptide sequence
			K.NIILTRINPMIR.D + Oxidation (M)
			K.DPLSFDGMADTEVERR.L
			R.MLHENRRPPK.E
			R.SVSATETSADVLHGIAIK.C
			R.SVSATETSADVLHGIAIKCGAK.V
			R.FSVEAWLSNQK.I
			R.DETALPVSSRPTDPRLEGSMR.H
26	gi|15341050	maturase K	K.DISCLHLLR.F
			K.DISCLHLLRFFLYEYCNSVIIIK.R
			R.VYFYLK.L
			R.GTFLLMTKLK.Y
			K.KFETLVPIMPMIGSLAK.A + Oxidation (M)
			K.FCNVLGHPISKPAWADLSDSDIIR.R
			R.FGRMCR.N + Oxidation (M)
	Function unknown and hypothetical proteins		
18	gi|125547814	hypothetical protein OsI_15422	R.TLFRTSGDVVAR.M
			R.TSGDVVAR.M
			R.DRLVESYTWSQMLFYEEGLALTR.I
			R.LVESYTWSQMLFYEEGLALTR.I + Oxidation (M)
			K.TSTVQLLCVSTTVGR.G
			K.LDSLIEDEWRTMNHAR.Y
			K.DAYTFSTHLQEIVRSLFVNPIPI.-

Table S2. *Cont.*

Spot [a]	NCBI accession [b]	Protein identity [c]	Peptide sequence
22	gi|296087931	unnamed protein product	-.MTHSQK.D + Oxidation (M)
			K.DVAYHAEDLLDEIATEALR.C
			K.AWNWEKVSTWVK.V
			K.IVVTSRSETAAK.I
			R.SETAAKIMR.A + Oxidation (M)
			K.DYEFDKEK.L
			K.LQKISDK.A
			K.AVVFETFESVK.R
			R.LPESICCLCNLQTMMLSKCR.C + 2 Oxidation (M)
			R.YLDISGSNSLKEMPNDIDQLK.S + Oxidation (M)
			K.LEISKMENVVGVEDALQAHMK.D + 2 Oxidation (M)

[a] Assigned spot number as indicated in Figure 2C and Table 2; [b] Database accession numbers according to NCBInr; [c] The name of the proteins identified by MALDI-TOF MS.

© 2014 by the authors; licensee MDPI, Basel, Switzerland. This article is an open access article distributed under the terms and conditions of the Creative Commons Attribution license (http://creativecommons.org/licenses/by/3.0/).
